# Supplementary material for: A Pleiotropy-Informed Bayesian False Discovery Rate Adapted to a Shared Control Design Finds New Disease Associations From GWAS Summary Statistics
Source: PLoS Genet. 2015 Feb 6;11(2):e1004926. doi: 10.1371/journal.pgen.1004926 (PMC4450050; doi:10.1371/journal.pgen.1004926)
Supplement: S1 Text — (PDF) [file pgen.1004926.s001.pdf]

# A Pleiotropy-Informed Bayesian False Discovery Rate adapted to a Shared Control Design Finds New Disease Associations From GWAS Summary Statistics
# Supplementary Material

James Liley    Chris Wallace

November 8, 2014

## Contents

**A Robustness of normality assumption in estimating distribution of effect sizes** **2**

**B An upper bound for the expected FDR for a union of regions each with known FDR** **8**

**C Overestimation of expected quantile $P(P_i \leq p_i|P_j \leq p_j)$ by raw p value** **13**

**D Maximum possible overestimation of expected quantile $P(P_i \leq p_i|P_j \leq p_j)$** **21**

# A Robustness of normality assumption in estimating distribution of effect sizes

The computation of cFDR with shared control groups requires an estimate of the distribution of true effect sizes $\eta$ in the conditional phenotype; that is, the $z$ values which would be observed if the MAFs in controls and cases exactly matched the true MAFs in the relevant populations. We assume that the values $\eta$ are instances of a random variable $H$.

For any distribution of effect sizes which is non-increasing for positive effect sizes and non-decreasing for negative effect sizes, the true cFDR is less than the 'naive' cFDR obtained from applying the Andreasson formula without adjustment (supplementary material, theorem 2). That the distribution of true effect sizes should have this profile is essentially the assumption that 'small' effect sizes are more frequent than 'large' effect sizes in polygenic phenotypes, a hypothesis suggested by recent large GWAS [1].

In our algorithm, we assume the true effect sizes $\eta$ follow a mixture distribution, being 0 with probability $\pi_0$ and following a normal distribution with mean 0 and standard deviation $\sigma$ with probability $1 - \pi_0$. This is largely for computational convenience, as it allows a simplification of a triple integral to a double (Methods). However, the formula can be applied for any distribution of $H$.

The information about the distribution of $H$ is incorporated into the formula for $\widehat{cFDR}$ through the expression $Pr(P_i \leq p_i | P_j \leq p_j, H_0^{(i)})$. As shown in the supplementary material (theorems 1 and 2) this is generally larger than $p_i$ for most conceivable distributions of $H$.

If the true distribution of $H$ is different to that assumed, the estimate of $Pr(P_i \leq p_i | P_j \leq p_j, H_0^{(i)})$ will be incorrect. Overestimation of $Pr(P_i \leq p_i | P_j \leq p_j, H_0^{(i)})$ is a less serious problem, as it will lead to a (conservative) overestimate of $\widehat{cFDR}$. Since $\widehat{cFDR}$ is a systematically conservative estimate of $cFDR$ anyway, the expected $\widehat{cFDR}$

using the incorrect distribution of $H$ will still provide an upper bound on the true $cFDR$ $(= Pr(H_0^{(i)}|P_i \leq p_i, P_j \leq p_j))$, although we would expect to lose power.

If, however, an incorrect assumption on the distribution of $H$ leads to an underestimation of $cFDR$, the estimate may no longer be conservative, meaning that the expected value of $\widehat{cFDR}$ may not be a true upper bound on $\widehat{cFDR}$. While it is impossible to know the true distribution of $H$, we show in theorem 3 that the distribution of $\eta$ for which $Pr(P_i \leq p_i|P_j \leq p_j, H_0^{(i)})$ is highest is the degenerate distribution at 0; that is, if $\pi_0 = 1$, so the worst the underestimate can be is in the case in which all SNPs are null for the conditional phenotype.

For a SNP with p values $(p_i, p_j)$ for two phenotypes $i$, $j$, the percentage difference in $Pr(P_i \leq p_i|P_j \leq p_j, H_0^{(i)})$ when using the normal approximation compared to the 'true' distribution of $H$ is equal to the percentage difference in $\widehat{cFDR}(p_i|p_j)$ and indicates the range of potential $\widehat{cFDR}$ cutoffs for which the normal approximation will lead to a different classification of the SNP. However, this does not account for the distribution of values of $(p_i, p_j)$; clearly some values of $p_j$ are far more likely than others, and in general the more likely values of $(p_i, p_j)$ tend to correspond to smaller errors. An implication of this is that although an incorrect assumption of the distribution of $H$ could theoretically lead to a 20% over- or underestimation of the true $\widehat{cFDR}$, a much smaller error may be observed in the actual number of SNPs for which $\widehat{cFDR}$ is less than some threshold.

To show this, we simulated observed effect sizes for 10000 SNPs for a principal phenotype $i$ and a conditional phenotype $j$. The $Z$ scores for the conditional phenotype were distributed according to various non-Gaussian distributions, and the $Z$ scores for the principal phenotype were distributed as $N(0, 5)$, in order to generate values with a reasonable range. $Z$ scorese were correlated with $\rho = 0.3$, corresponding to partial sharing of controls. Values of $\widehat{cFDR}$ were computed using first the true distribution of effect sizes for

the conditional phenotype and second a normal approximation. We compared the number of SNPs reaching a range of a range of thresholds for $\widehat{cFDR}$.

Figures 1A-D show the amount by which $Pr(P_i \leq p_i | P_j \leq p_j, H_0^{(i)})$ is overestimated or underestimated using our technique for various distributions of $H$. The leftmost panels show the percentage error which arises for possible values $(p_i, p_j)$ (p values for the principal and conditional phenotype respectively), and the middle pattern shows the number of SNPs with $\widehat{cFDR}$ less than a given cutoff with cFDR calculated using either the true distribution or the normal approximation of $H$. The rightmost panel shows the true distribution of $H$ and the normal approximation to it.

When $H$ has a bimodal distribution, the error is almost universally below 5%. In general, if the true distribution of $H$ follows a heavy-tailed distribution, such as the T-distribution, then because our approximation comparatively under-weights extreme $H$ values, which would otherwise lower the estimate of $Pr(P_i \leq p_i | P_j \leq p_j, H_0^{(i)})$, in favour of values nearer zero, our approximation tends to overestimate rather than underestimate $Pr(P_i \leq p_i | P_j \leq p_j, H_0^{(i)})$, which, as discussed, is favourable. Except for very pathological distributions, the overestimation or underestimation is rarely greater than 20%. For all tested distributions, the effect on the number of SNPs with $\widehat{cFDR}$ less than a cutoff is negligible.

Figure 1 E simulates a scenario in which all SNPs are null for the conditional phenotype, but in which our estimated distribution of $H$ has parameters $pi_0 = 0.9$, $\sigma = 2$. In practice, our E-M algorithm for estimating the parameters of the distribution will generally return $\pi_0 = 1$, meaning such a scenario is unlikely to occur if the observed values of $P_j$ do not differ substantially from their underlying distribution. This figure indicates the maximum possible underestimation of $Pr(P_i \leq p_i | P_j \leq p_j, H_0^{(i)})$, given incorrectly estimated values of $\pi_0$ and $\sigma$. Notably, it is highest when both $p_i$ and $p_j$ are very low.

Although the left-hand panel of figure 1 E demonstrates that there is potential for major underestimation of the expected quantile, the middle panel shows that in this scenario the misestimation has minimal effect on the number of SNPs reaching various $\widehat{cFDR}$ thresholds. This is because the major underestimation occurs at very low $p_j$ values, which are very rare under the true distribution $H = 0$.

We conclude from these results that a normal approximation to the distribution of $H$ is reasonable in most cases.

**1A. Correct uniform distribution**

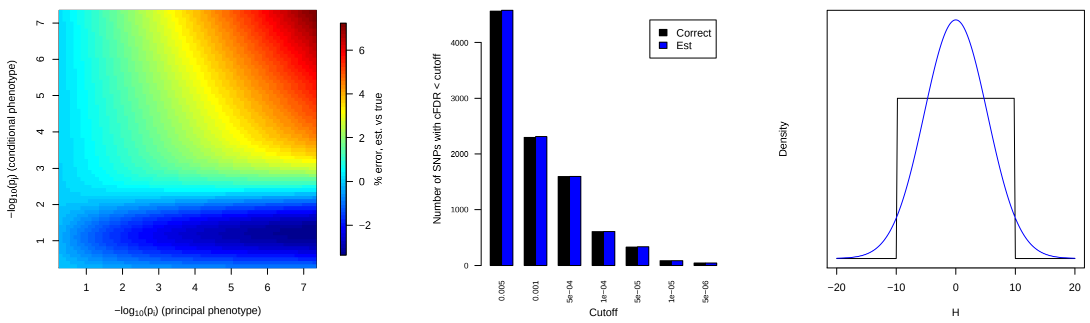


**1B. Correct T distribution, 5DF**

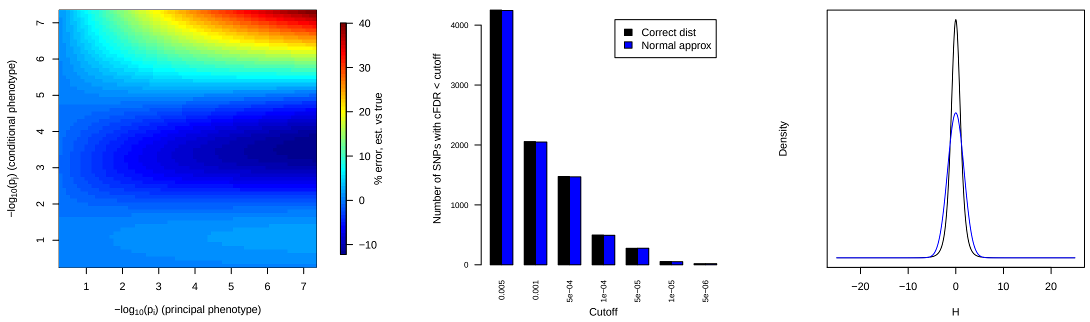


**1C. Correct bimodal distribution**

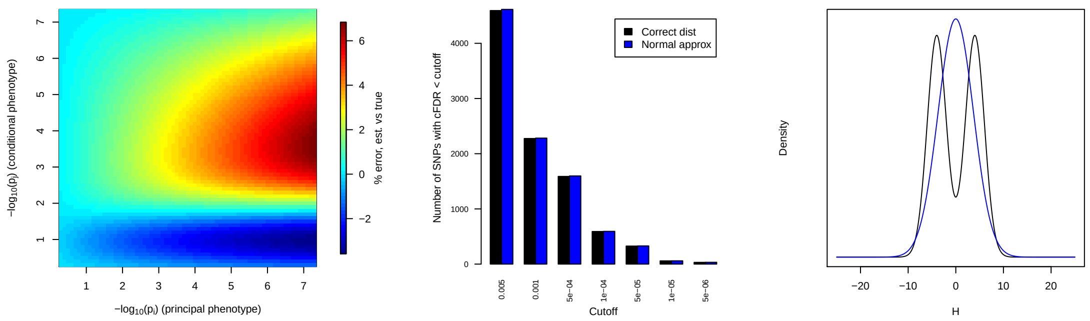


Continued on next page.

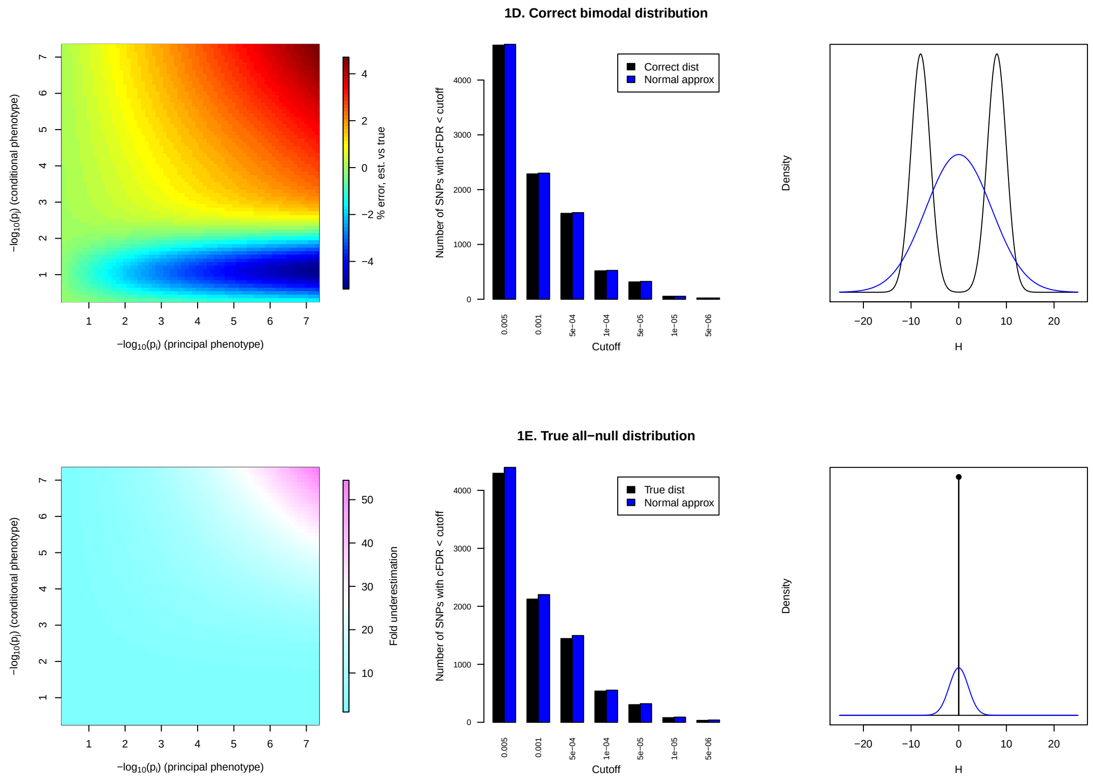


Figure 1: Figures showing the effect of incorrectly estimating the distribution of conditional effect sizes. If the distribution of 'true' effect sizes for the conditional phenotype (the effect sizes which would be observed in a study of equal size if the observed MAFs exactly matched population MAFs) is incorrectly assumed, the value $Pr(P_i \leq p_i | P_j \leq p_j, H_0^{(i)})$ (proportional to $\widehat{cFDR}$) will be incorrectly calculated. These figures demonstrate a range of possible distributions for the true effect sizes, with an assumed value of $\pi_0 = 0.9$ in each case. In all cases, we simulated $10^5$ samples from the 'true' distribution and used our E-M algorithm to find an approximating $\pi_0$ and $\sigma$. For panels A-D, the right-hand plot shows the 'true' conditional effect size distribution (uniform, T (df=5), and bimodal with near and far peaks respectively) and our approximation and the left-hand plot the percentage error in the estimate. Note that colours correspond to different values in the different graphs due to difficulties in scaling. Panel E shows the maximum fold underestimation of cFDR, which occurs the true effect sizes are all zero. The 'estimated' distribution here is not what would be estimated by our E-M algorithm (which would find the correct distribution in this case) but a simulated incorrect distribution.

# B An upper bound for the expected FDR for a union of regions each with known FDR

Let $\mathscr{P} = P_i \times P_j$ denote the unit square, with the x co-ordinate of a point in $\mathscr{P}$ corresponding to a p value for the principal phenotype and the y co-ordinate corresponding to the p value for the conditional phenotype.

For a region $X$ of $\mathscr{P}$ , define the random variable $Q_X$ as the ratio of $V_X$, the number of SNPs in $X$ which are null for the principal phenotype, to $R_X$, the total number of SNPs in $X$.

We prove here that if $L$ is the region of the space $\mathscr{P}$ in which $\widehat{cFDR}(p_i|p_j) \leq \alpha'$, and $M^*$ is some rectangle with vertices $(0,0), (p_i^*, 0), (p_i^*, p_j^*), (0, p_j^*)$ contained in $L$, then

$$E(Q_L) \leq \alpha^* \frac{v(L)}{v(M^*)} \tag{1}$$

where $v(L)$ and $v(M^*)$ are the expected number of null SNPs in $L$ and $M^*$, and $\alpha^* = \widehat{cFDR}(p_i^*, p_j^*)$.

We begin with the following lemma.

**Lemma 1.** *If we consider the set $S$ of SNPs for which $P_j \leq p_j^*$ and reject the null hypothesis for all SNPs in $S$ for which the value of $\widehat{cFDR}(p_i|p_j^*)$ is less than $\alpha$, then $E(Q) \leq \alpha$*

*Proof.* We employ Theorem 1 in Benjamini and Hochberg [2].

Denote by $p_{i1}, p_{i2}...p_{iN}$ the p values for the principal phenotype for SNPs in S, and $P_i$ the random variable of which these values are instances. For convenience, assume $p_{i1} \leq p_{i2} \leq ...p_{iN}$. Our p value adjustment maps $p_{ik}$ to $p'_{ik} = Pr(P_i \leq p_{ik}|P_j \leq p_j^*, H_0^{(i,k)})$, where $H_0^{(i,k)}$ is the null hypothesis for SNP k for the principal phenotype. Denote by $P'_i$ the random variable of which the values $p'_{ik}$ are instances.

We note firstly that as the conditioning is the same for all $p_{ik}$, we have $p'_{i1} \leq p'_{i2} \leq ...p_{iN}$. We also note that with shared controls, $P_i|H_0^{(i)} \nsim U(0,1)$, but $P'_i|H_0^{(i)} \sim U(0,1)$; that is, the distribution of $P_i$ for null SNPs is non-uniform, but the distribution of $P'_i$ for null SNPs is uniform. Therefore the values $p'_{ik}$ behave like p values for null SNPs. We have

$$\widehat{cFDR}(p_{ik}|p^*_j) = p'_{ik}\frac{N}{k} \tag{2}$$

to which the similarity to Benjamini and Hochberg's method is obvious. Applying their theorem yields the required result.

□

As a corollary, we note that the SNPs whose null hypotheses we reject are those within the rectangle $R*$ with vertices $(0,0),(p^*_i,0),(p^*_i,p^*_j),(0,p^*_j)$, where $p^*_i$ is the largest value of $\{p_{ik}\}$ for which $\widehat{cFDR}(p_{ik}|p^*_j) \leq \alpha$.

**Corollary 1.** *If* $R^*$ *is a rectangle with vertices* $(0,0),(p^*_i,0),(p^*_i,p^*_j),(0,p^*_j)$, *and* $\widehat{cFDR}(p^*_i|p^*_j) = \alpha'$, *then* $E(Q_{R^*}) \leq \alpha'$

It is tempting to assume that corollary 1 extends to the region $L$ as well as rectangles $R$. Unfortunately, this does not hold. However, a bound on the $E(Q_L)$ can be obtained by considering the distributions of p value pairs of null and non-null SNPs.

For reasonable values of $\alpha'$, $L$ can be expected to contain a strip on the leftmost border of $\mathscr{P}$, corresponding to $P_i \leq 5 \times 10^{-8}$, but not contain any points on the rightmost border $(1,0)$. Therefore, the region will be bounded by the lines $p_i = 0$, $p_j = 0$, $p_j = 1$, and a curve $p_j = l(p_i)$.

Let $M^* = M(p'_i, p'_j)$ be the largest rectangle by area of the form above contained within $L$, and $\alpha^*$ the $\widehat{cFDR}$ at the upper right vertex.

Assume that among all SNPs in $\mathscr{P}$ we have $m_0$ which are null for phenotype $i$, and $m_1$ which are non-null. Denote by $f_0(p_i, p_j)$ the *pdf* of null p-values $P_i$ and $P_j$. For any closed simple region $X$, the expected number of null SNPs whose P value pairs fall within $X$ is $m_0 v(X)$, where $v(X)$ denotes the integral of $f_0$ over $X$. This is close to the area of $X$; however, because we expect that $P_j$ has a non-uniform distribution at null SNPs, and because $P_i$ and $P_j$ have some dependence due to shared controls, the distribution will be somewhat skewed toward $(0, 0)$. We discuss the estimation of $f_0$ in section B.

Likewise, if we denote the density of the distribution of non-null SNPs by $f_1(p_i, p_j)$, the expected number of non-null SNPs in $X$ is given by

$$m_1 \int_X f_1(p_i, p_j) dp_i dp_j$$

Hence

$$\begin{aligned} E(Q_X) &= \frac{m_0 v(X)}{m_0 v(X) + m_1 \int_X f_1(p_i, p_j) dp_i dp_j} \\ &= \frac{v(X)}{v(X) + \frac{m_1}{m_0} \int_X f_1(p_i, p_j) dp_i dp_j} \end{aligned} \tag{3}$$

and specifically, by corollary 1

$$E(Q_{M'}) = \frac{v(M')}{v(M') + \frac{m_1}{m_0} \int_{M'} f_1(p_i, p_j) dp_i dp_j} \leq \alpha' \tag{4}$$

From the definition of $M$ and the above;

$$\int_L f_1(p_i, p_j) dp_i dp_j \geq \int_{M'} f_1(p_i, p_j) dp_i dp_j \geq v(M') \frac{m_0}{m_1} (\frac{1}{\alpha'} - 1), \tag{5}$$

thus

$$\begin{aligned} E(Q_L) &= \frac{v(L)}{v(L) + \frac{m_1}{m_0}\int_L f_1(p'_i, p'_j) dp'_i dp'_j} \\ &\leq \frac{v(L)}{v(L) + v(M')(\frac{1}{\alpha'} - 1)} \\ &\leq \frac{v(L)}{v(M')}\alpha' \end{aligned} \tag{6}$$

giving a bound on the expected value of the false discovery rate for the region $L$.

## Estimation of the distribution of p values for the principal phenotype across SNPs null for the conditional phenotype

The distribution of p values for the principal phenotype across SNPs null for the conditional phenotype can be derived from the distribution of $Z$ scores given in the Methods section of the main paper.

$$(Z_i, Z_j | H_0^{(i)}) \sim \begin{cases} N(\left(\begin{smallmatrix} 0 \\ 0 \end{smallmatrix}\right), \left(\begin{smallmatrix} 1 & \rho \\ \rho & 1 \end{smallmatrix}\right)), & p = \pi_0^{(j)} \\ N(\left(\begin{smallmatrix} 0 \\ 0 \end{smallmatrix}\right), \left(\begin{smallmatrix} 1 & \rho \\ \rho & 1+\sigma^2 \end{smallmatrix}\right)), & p = 1 - \pi_0^{(j)} \end{cases} \tag{7}$$

While we previously computed a distribution $(Z_i, Z_j | H_0^{(i)})$ in order to compute the expected quantile, here our motivation differs. When computing the expected quantile, the motivation was to determine what the expected distribution for $(Z_i, Z_j | H_0^{(i)})$ would be *if* the SNP were null for the principal phenotype; in this section, we aim to find the expected distribution *only* for truly null SNPs. For this reason, while we fitted the earlier model to *all* values $Z_j$; here, we only fit the model to a subset of values $Z_j$ which we expect to correspond to null SNPs.

We fit our model to the $Z_j$ values for the SNPs corresponding to $P_i \geq 0.5$. Because

we expect true effect sizes to be independent at SNPs which are null for the principal phenotype, this will be a representative sample of the distribution of $Z_j$ for these SNPs. We expect that some non-null SNPs will also have $P_i \geq 0.5$; however, the proportion of such SNPs will be small, and their inclusion would be expected to lead to an estimate of the distribution of $P_j$ biased toward low values, affecting the integral of $f_0$ over $L$ more than the integral over $X$ and making our estimate conservative.

We assume $Pr(P_j \leq p_j | P_i \geq 0.5, H_0^{(i)}) \approx Pr(P_j \leq p_j | H_0^{(i)})$ and fit $\pi_0'$, $\sigma'$ to the model

$$Z_j | H_0^{(i)} \sim Z_j | P_i \geq 0.5, H_0^{(i)} \sim \pi_0' N(0,1) + (1-\pi_0')N(0,\sigma'^2) \tag{8}$$

using expectation/maximisation.

Defining $f_{\rho,\pi_0,\sigma}(z_i, z_j)$ as

$$f_{\rho,\pi_0',\sigma'}(z_i,z_j) = \pi_0' N_{\binom{0}{0},\left(\begin{smallmatrix}1 & \rho \\ \rho & 1\end{smallmatrix}\right)}(z_i,z_j) + (1-\pi_0')N_{\binom{0}{0},\left(\begin{smallmatrix}1 & \rho \\ \rho & 1+\sigma'^2\end{smallmatrix}\right)}(z_i,z_j) \tag{9}$$

the *pdf* of p value pairs $(P_i, P_j) = (2\ \Phi(-|Z_i|), 2\ \Phi(-|Z_j|))$ can then be written as

$$\begin{aligned} pdf(p_i,p_j) = f_0(p_i,p_j) = \big(& f_{\rho,\pi_0',\sigma'}(-\Phi^{-1}(\frac{p_i}{2}), -\Phi^{-1}(\frac{p_j}{2})) \\ &+ f_{\rho,\pi_0',\sigma'}(\Phi^{-1}(\frac{p_i}{2}), -\Phi^{-1}(\frac{p_j}{2})) \\ &+ f_{\rho,\pi_0',\sigma'}(-\Phi^{-1}(\frac{p_i}{2}), \Phi^{-1}(\frac{p_j}{2})) \\ &+ f_{\rho,\pi_0',\sigma'}(-\Phi^{-1}(\frac{p_i}{2}), \Phi^{-1}(\frac{p_j}{2}))\big) \\ &\times \frac{\pi}{2} exp(\frac{1}{2}(\Phi^{-1}(\frac{p_i}{2})^2 + \Phi^{-1}(\frac{p_j}{2})^2)) \end{aligned} \tag{10}$$

where $\Phi(z)$ denotes as usual the normal *cdf* at $z$, and $\Phi^{-1}(p)$ its inverse at $p$. If the upper right vertex of $M_*$ is on the line $P_j = 1$, then the integral of this *pdf* over the rectangle $M^*$ is simply the area of $M^*$, as it corresponds to the area of the marginal *pdf* for $P_i$,

which is $U(0,1)$ by assumption. The integral of the *pdf* over $L$ is easiest to obtain by integrating expression 9 over the analogue of $L$ on the plane $Z_i \times Z_j$. If $\sigma' = 1$ then the *pdf* is identically 1 and this integral is the area of $L$.

# C Overestimation of expected quantile $P(P_i \le p_i | P_j \le p_j)$ by raw p value

**Theorem 1.** *Given two bivariate normal random variables* $(Z_i, Z_j)$ *with means* $(0,0)$ *and covariance matrix* $\begin{pmatrix} 1 & \rho \\ \rho & 1+\sigma^2 \end{pmatrix}$, *define* $P_i = 2\Phi(-|Z_i|)$ *and* $P_j = 2\Phi(-|Z_j|)$ *(the p values associated with* $Z_i$ *and* $Z_j$*). Then for any* $p_i$, $p_j \ge 0$

$$Pr(P_i \le p_i | P_j \le p_j) \ge p_i \tag{11}$$

*with equality if and only if* $\rho = 0$, $p_i = 0$, *or* $p_j = 0$.

*Proof.* Define $z_i = \Phi^{-1}(p_i/2)$, $z_j = \Phi^{-1}(p_j/2)$, and let

$$\begin{aligned} f_\sigma(x) &= \frac{1}{\sqrt{2\pi(1+\sigma^2)}} exp(-\frac{1}{2(1+\sigma^2)}x^2) \\ f_\sigma(x,y,\rho) &= \frac{1}{2\pi\sqrt{1+\sigma^2-\rho^2}} exp(-\frac{1}{2(1+\sigma^2-\rho^2)}((1+\sigma^2)x^2 - 2\rho xy + y^2)) \end{aligned} \tag{12}$$

the univariate and bivariate normal *pdf*s corresponding to the distributions of $Z_j$ and $(Z_i, Z_j)$ respectively.

The statement to be proved is equivalent to

$$
\begin{aligned}
& Pr(P_i \leq p_i, P_j \leq p_j) \geq p_i p_j \\
\Leftrightarrow \quad & Pr(|Z_i| \geq z_i, |Z_j| \geq z_j) \geq Pr(|Z_i| \geq z_i) Pr(|Z_j| \geq z_j) \\
\Leftrightarrow \quad & \iint_{|x|\geq z_i, |y|\geq z_j} f_\sigma(x,y,\rho)dxdy \geq \int_{|x|\geq z_i} f_0(x)dx \int_{|y|\geq z_j} f_\sigma(y)dy \qquad (13) \\
& = \iint_{|x|\geq z_i, |y|\geq z_j} f_\sigma(x,y,0)dxdy
\end{aligned}
$$

Due to the symmetry of the normal distribution and the four disjoint regions defined by $\{|x| \geq z_i, |y| \geq z_j\}$, we may rewrite both integrals over the connected region in $\{\mathbb{R}^+\}^2$ defined by $\{x \geq z_i, y \geq z_j\}$:

$$\iint_{x\geq z_i, y\geq z_j} f_\sigma(x,y,\rho) + f_\sigma(x,y,-\rho) - 2f_\sigma(x,y,0)dxdy \geq 0 \qquad (14)$$

Rewriting $f_\sigma(x,y,\rho)$ as

$$\frac{1}{2\pi\sqrt{1+\sigma^2-\rho^2}} exp(-\frac{1+\sigma^2}{2(1+\sigma^2-\rho^2)}(x-\frac{\rho}{1+\sigma^2})^2) exp(-\frac{1}{2(1+\sigma^2)}y^2) \qquad (15)$$

and noting that

$$\int_{z_i}^{\infty} exp(-\frac{1+\sigma^2}{2(1+\sigma^2-\rho^2)}(x-\frac{\rho}{1+\sigma^2})^2 y)dx = \sqrt{2\pi\frac{(1+\sigma^2-\rho^2}{1+\sigma^2}})\Phi(\frac{-z_i+\frac{\rho}{1+\sigma^2}y}{\sqrt{\frac{1+\sigma^2-\rho^2}{1+\sigma^2}}})$$

we can rewrite inequality 15, after removing the common denominator $2\sqrt{2\pi(1+\sigma^2)}$, as

$$\int_{z_j}^{\infty} \big(\Phi(\frac{-z_i+\frac{\rho}{1+\sigma^2}y}{\sqrt{\frac{1+\sigma^2-\rho^2}{1+\sigma^2}}}) + \Phi(\frac{-z_i-\frac{\rho}{1+\sigma^2}y}{\sqrt{\frac{1+\sigma^2-\rho^2}{1+\sigma^2}}}) - 2\Phi(-z_i)\big) exp(-\frac{1}{2(1+\sigma^2)}y^2)dy > 0 \qquad (16)$$

If $z_j = 0$, then the region integrated over in 13 is two vertical strips defined by $|x| \geq z_i, y \in$

$\mathbb{R}$. The integral of $f_\sigma(x, y, \rho)$ over this region is the integral of the marginal of $Z_i$ over $|x| \geq z_i$, and is hence independent of $\rho$. Thus, in this case, the integral of $f_\sigma(x, y, \rho) + f_\sigma(x, y, -\rho) - 2f_\sigma(x, y, 0)$ over this region is 0. The same clearly holds if $z_i = 0$ or $\rho = 0$. Henceforth, we will assume $\rho > 0$.

Define

$$g(y) = \Phi(\frac{-z_i + \frac{\rho}{1+\sigma^2}y}{\sqrt{\frac{1+\sigma^2-\rho^2}{1+\sigma^2}}}) + \Phi(\frac{-z_i - \frac{\rho}{1+\sigma^2}y}{\sqrt{\frac{1+\sigma^2-\rho^2}{1+\sigma^2}}}) - 2\Phi(-z_i)$$
$$h(y) = g(y)exp(-\frac{1}{2(1+\sigma^2)}y^2) \tag{17}$$

Because $\Phi$ is monotonically increasing and $(1+\sigma^2-\rho^2)/(1+\sigma^2) < 1$ we have $g(0) < 0$. As $y \to \infty$, the first term in $g$ tends to 1, and the second to 0. As $\Phi(-z_i) < 0.5$, $g$ therefore tends to the finite positive value $1 - 2\Phi(-z_i)$, and hence $h(y)$ tends to 0 from above.

We have

$$g'_{z_i}(y) = C_0\big(exp(-\frac{(1+\sigma^2)(-\frac{\rho}{1+\sigma^2}y + z_i)^2}{2(1+\sigma^2-\rho^2)}) - exp(-\frac{(1+\sigma^2)(\frac{\rho}{1+\sigma^2}y + z_i)^2}{2(1+\sigma^2-\rho^2)})\big) \tag{18}$$

for a constant $C_0$, which can only be 0 if the exponentiated terms are equal; that is, $y = 0$ or $z_i = 0$. Given that $h$ is asymptotically positive and $g(0) < 0$, $g$ is monotonically increasing on $\mathbb{R}^+$, and is 0 at exactly one positive value $y_0$. Because the sign of $h$ is the sign of $g$, $h(y)$ is likewise 0 if and only if $y = y_0$.

For $y > y_0$, $g(y)$ is positive, so $h(y)$ is uniformly positive across the region of integration, and hence the integral 16 is positive for $z_j > y_0$. Because the integral of $h$ over the positive

reals is 0 we have, for any $z_j \leq y_0$

$$\begin{aligned}\int_{z_j}^{\infty} h(y)dy &= \int_{z_j}^{y_1} h(y)dy + \int_{y_1}^{\infty} h(y)dy \\ &> \int_{0}^{y_1} h(y)dy + \int_{y_1}^{\infty} h(y)dy \\ &= \int_{0}^{\infty} h(y)dy \\ &= 0 \end{aligned} \tag{19}$$

as required.

□

**Corollary 2.** *Suppose a SNP is null for two phenotypes $i$ and $j$, and GWAS are performed for $i$ and $j$ sharing some or all controls. Let $p_i$ be the p value obtained at the SNP for phenotype $i$, $p_j$ be the p value for phenotype $j$, $P_i$, $P_j$ the random variables from which $p_i$ and $p_j$ are drawn, and $H_0^{(i)}$ the null hypothesis for the SNP for phenotype $i$. Then $p_i$ underestimates the probability $Pr(P_i \leq p_i | P_j \leq p_j, H_0^{(i)})$, and hence leads to an unpredictably biased and usually falsely low estimate of $cFDR$.*

*Proof.* Due to a result of Zaykin et al [3,4] the sharing of controls between studies induces a positive correlation between Z scores. Applying the theorem with $\sigma = 0$ yields the result. The estimated $cFDR$ is computed by dividing the quantity $Pr(P_i \leq p_i | P_j \leq p_j, H_0^{(i)})$ by an estimate of $Pr(P_i \leq p_i | P_j \leq p_j)$. As discussed in the methods section, the estimate is systematically slightly conservative (a slight overestimate) as the quantity $Pr(H_0^{(i)} | P_j \leq p_j)$ is assumed to be 1. This conservatism is lost if the estimate of $Pr(P_i \leq p_i | P_j \leq p_j, H_0^{(i)})$ is systematically low (as is the case if $p_i$ is used as the estimator); the assertion that $E(\widehat{cFDR}) \geq \widehat{cFDR}$ no longer holds. □

**Corollary 3.** *Suppose a GWAS is performed for some phenotype $j$. For each SNP $s$ define*

*$\eta_s$ as the Z score which would have been obtained in the GWAS if the allele frequencies for that SNP in the case and control group exactly matched the allele frequencies in the general case and control populations. Consider the values $\eta_s$ as observations of a continuous random variable $H$ and suppose that*

$$H \sim \begin{cases} 0, & p = \pi_0^{(j)} \\ N(0, \sigma^2), & p = 1 - \pi_0^{(j)} \end{cases} \tag{20}$$

*for some $\pi_0, \sigma$. Suppose another GWAS is performed for some other phenotype $i$, sharing some or all controls with phenotype $j$. Let $p_i$ and $p_j$ be the $p$ values obtained in these two studies for a randomly chosen SNP, and $P_i$, $P_j$ the random variables associated with $p_i$ and $p_j$. Define $H_0^{(i)}$ as the hypothesis that the SNP is null for phenotype $i$. Then $p_i$ (equal to $Pr(P_i \leq p_i | H_0^i)$) underestimates $Pr(P_i \leq p_i | P_j \leq p_j, H_0^{(i)})$ , and hence leads to an unpredictably biased and usually falsely low estimate of $cFDR$.*

*Proof.* As in the section 'Computation of conditional False Discovery Rate', define

$$\begin{aligned} \Lambda_{(\rho,\sigma^2)}(z_i, z_j) &= \iint_{|x|>|z_i|,|y|>|z_j|} N_{\left(\begin{smallmatrix}0\\0\end{smallmatrix}\right),\left(\begin{smallmatrix}1 & \rho\\ \rho & 1+\sigma^2\end{smallmatrix}\right)}(x, y)dxdy \\ \lambda_{\sigma^2}(z_j) &= \int_{|y|>|z_j|} N_{(0,1+\sigma^2)}(y)dy \end{aligned} \tag{21}$$

By the results obtained in the same section, the distribution of H implies that

$$Pr(P_i \leq p_i | P_j \leq p_j, H_0^{(i)}) = \frac{\pi_0^{(j)} \Lambda_{(\rho,0)}(z_i, z_j) + (1 - \pi_0^{(j)})\Lambda_{(\rho,\sigma^2)}(z_i, z_j)}{\pi_0^{(j)} \lambda_0(z_j) + (1 - \pi_0^{(j)})\lambda_{\sigma^2}(z_j)} \tag{22}$$

for some $\rho > 0$. From corollary 2 we have $\Lambda_{(\rho,0)}(z_i, z_j)/\lambda_0(z_j) > p_i$ and from theorem 1 we have $\Lambda_{(\rho,\sigma^2)}(z_i, z_j)/\lambda_{\sigma^2}(z_j) > p_i$. The result follows. □

**Theorem 2.** *Define $i$, $j$, $(Z_i, Z_j)$, $(p_i, p_j)$, $(P_i, P_j)$, $(p_i, p_j)$, $\eta$, and $H$ as for corollary 3, but suppose $H$ is distributed according to some unknown distribution function $k(x)$ which is non-increasing on $\mathbb{R}^+$. Then $p_i$ underestimates $Pr(P_i \leq p_i | P_j \leq p_j, H_0^{(i)})$.*

*Proof.* We prove the statement for $H \sim U(0, \eta_{max})$ (the uniform distribution) for any $\eta_{max}$. Because any decreasing function can be arbitrarily closely approximated as a linear combination of such functions with non-negative coefficients, we conclude the result.

We may assume without loss of generality that $\eta$ is nonnegative. Defining $k$ again as the *pdf* of $H$, we have

$$Pr(P_i \leq p_i | P_j \leq p_j, H_0^{(i)}) = \frac{Pr(P_i \leq p_i, P_j \leq p_j, H_0^{(i)})}{Pr(P_j \leq p_j, H_0^{(i)})} = \frac{\int_0^\infty Pr(P_i \leq p_i, P_j \leq p_j | H_0^{(i)}, H = \eta) k(\eta) d\eta}{\int_0^\infty Pr(P_j \leq p_j | H_0^{(i)}, H = \eta) k(\eta) d\eta} \tag{23}$$

so the statement is equivalent to proving that

$$\int_0^\infty k(\eta) \Big( \iint_{|x| > z_i, |y| > z_j} f_0(x, y - \eta, \rho) - f_0(x, y - \eta, 0) dx dy \Big) d\eta > 0 \tag{24}$$

For brevity, let $q(x, y, \eta) = f_0(x, y - \eta, \rho) - f_0(x, y - \eta, 0)$. Assume that $k(\eta)$ has the form

$$k(\eta) = \begin{cases} \frac{1}{\eta_{max}} \texttt{ if } \eta \leq \eta_{max} \\ 0 \texttt{ if } \eta > \eta_{max} \end{cases} \tag{25}$$

for some $\eta_{max}$. Then inequality 24 is equivalent to

$$\frac{1}{\eta_{max}} \int_0^{\eta_{max}} \Big( \iint_{|x| > z_i, |y| > z_j} q(x, y, \rho) dx dy \Big) d\eta \stackrel{\text{def}}{=} \frac{1}{\eta_{max}} I(\eta_{max}) > 0 \tag{26}$$

Because $f_0(x, y, \rho) = f_0(-x, -y, \rho)$ we have $q(x, y, \eta) = q(-x, \eta, y)$. Because of the sym-

metry of the function $q(x, y, \eta)$ about $y = \eta$, we have

$$\begin{aligned}
\int_0^\infty \iint_{|x|>z_i,|y|>z_j} q(x,y,\eta)dxdyd\eta &= \frac{1}{2}\int_{-\infty}^{\infty}\iint_{|x|>z_i,|y|>z_j} q(x,y,\eta)dxdyd\eta \\
&= \frac{1}{2}\int_{|y|>z_j}\iint_{|x|>z_i,\eta\in\mathbb{R}} q(-x,\eta,y)dxdyd\eta \\
&= \frac{1}{2}\int_{|y|>z_j}\iint_{|x|>z_i,\eta'\in\mathbb{R}} f_0(x,\eta',\rho) - f_0(x,\eta',0)dxd\eta dy \\
&= 0 \qquad (27)
\end{aligned}$$

setting $\eta' = \eta - y$, and again using the fact that the integral over the marginal is independent of $\rho$. Thus $I(\eta_{max}) \to 0$ as $\eta_{max} \to \infty$. Again using the symmetry of the function $q(x, y, \eta)$ about $y = \eta$, we can write

$$\iint_{|x|>z_i,|y|>z_j} q(x,y,\eta)dxdy =$$

$$\begin{cases} \frac{1}{2}(\iint_{|x|>z_i,|y|>\eta+z_j} q(x,y,0)dxdy - \iint_{|x|>z_i,|y|>\eta-z_j} q(x,y,0)dxdy), & \eta > z_j \\ \frac{1}{2}(\iint_{|x|>z_i,|y|>\eta+z_j} q(x,y,0)dxdy + \iint_{|x|>z_i,|y|>z_j-\eta} q(x,y,0)dxdy), & \eta < z_j \end{cases} \qquad (28)$$

From the second of these cases it is clear that the integrand of $I(\eta_{max})$ is always positive if $\eta_m ax \leq z_j$. If the integrand of $I(\eta_{max})$ (expression 28) is to be zero, we must have

$$\begin{aligned}
&\iint_{|x|>z_i,|y|>\eta+z_j} q(x,y,0)dxdy = \iint_{|x|>z_i,|y|>\eta-z_j} q(x,y,0)dxdy) \\
&\Leftrightarrow \int_{|y|>\eta+z_j}\int_{|x|>z_i} q(x,y,0)dydx = \int_{|y|>\eta-z_j}\int_{|x|>z_i} q(x,y,0)dydx \\
&\Leftrightarrow \int_{y>\eta+z_j} h(y)dy = \int_{y>\eta-z_j} h(y)dy \qquad (29)
\end{aligned}$$

using the notation $h(y)$ from theorem 1, and re-expressing the integrand over connected

regions. As discussed there, the integral of $h(y)$ is negative for $y$ less than some $y_0$ and positive thereafter, tending asymptotically to 0, so the integral of $h(y)$ from $y = z$ to $\infty$ is zero at $z = 0$, increases to a maximum at $z = x_0$, and decreases thereafter. If equality were to hold in the above, we would need the integral of $h(y)$ from $z$ to $\infty$ to be equal at two values of $z$ which are $2z_i$ apart. Because of the way the integral changes with $z$, the integral can only be zero for one value of $\eta$.

This implies that expression 28 is positive for $\eta < z_j$, is zero at some value $\eta = \eta_0$, and is negative thereafter. Given that $I(\eta_{max}) \to 0$ and is positive for $\eta_{max} < z_j$, it must be positive for all $\eta_{max}$. This proves the theorem for $k(\eta)$ of the form 25.

For any general *pdf* $k(\eta)$ which is nonincreasing on $\mathbb{R}^+$ and any $\epsilon > 0$ we may construct $k_0(\eta)$ as a linear sum of functions $k_{\eta_i}$ of the form 25:

$$\begin{aligned} k_0(\eta) &= \sum_i w_i k_{\eta_i}(\eta) \\ &\stackrel{\text{def}}{=} \sum_i w_i \begin{cases} \frac{1}{\eta_{max}} \ \texttt{if} \ \eta \leq \eta_i \\ 0 \ \texttt{if} \ \eta > \eta_i \end{cases} \end{aligned} \tag{30}$$

with nonnegative $w_i$, such that

$$\int_0^\infty |k(\eta) - k_0(\eta)| d\eta < \epsilon \tag{31}$$

Now

$$\iint_{|x|>z_i,|y|>z_j} f_0(x, y-\eta, \rho) - f_0(x, y-\eta, 0) dxdy \leq 2 \iint_{|x|>z_i,|y|>z_j} f_0(x, y-\eta, \rho) dxdy \leq 2 \tag{32}$$

so

$$\begin{aligned}
&\int_0^\infty k(\eta)\Big(\iint_{|x|>z_i,|y|>z_j} f_0(x,y-\eta,\rho)-f_0(x,y-\eta,0)dxdy\Big)d\eta \\
&-\int_0^\infty k_0(\eta)\Big(\iint_{|x|>z_i,|y|>z_j} f_0(x,y-\eta,\rho)-f_0(x,y-\eta,0)dxdy\Big)d\eta \\
&\leq 2\int_0^\infty k_0(\eta)d\eta \leq 2\epsilon
\end{aligned} \tag{33}$$

The second term in the LHS sum above is positive for any linear combination with at least one $w_i > 0$, as it is a linear combination of integrals of the form 26. Thus inequality 24 holds for any $k$ of the required form. □

*Remark* 1. For certain pathological distributions, it may be the case that $p_i$ does not underestimate $Pr(P_i \leq p_i|P_j \leq p_j)$. In general, however, most GWAS are across mostly null SNPs, so in general the distribution of $H$ (as per corollary 3) has a high mass at 0. In this case, the situation in corollary 2 is dominant, and in practice $p_i$ is almost always an underestimate of $Pr(P_i \leq p_i|P_j \leq p_j) \leq p_i$. Except in rare cases, $p_i \neq Pr(P_i \leq p_i|P_j \leq p_j)$, and it is not reasonable to use $p_i$ as an approximation in the current context if controls are shared between studies.

# D Maximum possible overestimation of expected quantile $P(P_i \leq p_i|P_j \leq p_j)$

**Theorem 3.** *Let $H$ be a random variable taking real values, and suppose that two random variables $Z_i$ and $Z_j$ are distributed as*

$$(Z_i, Z_j)|H = \eta \sim N\Big(\big(\begin{smallmatrix}0\\ \eta\end{smallmatrix}\big), \big(\begin{smallmatrix}1 & \rho\\ \rho & 1+\sigma^2\end{smallmatrix}\big)\Big) \tag{34}$$

*that is, normally distributed with mean* $(0, \eta)$ *and correlation matrix* $\begin{pmatrix} 1 & \rho \\ \rho & 1+\sigma^2 \end{pmatrix}$. *Let* $P_i = 2\Phi(-Z_i)$, $P_j = 2\Phi(-Z_j)$ *as usual, and* $(p_i, p_j)$ *be an instance of* $(P_i, P_j)$ *with* $0 < p_i, p_j < 1$. *Then across all distributions of H, the value of* $P(P_i \leq p_i | P_j \leq p_j)$ *is maximised when H is the degenerate distribution at 0; that is,* $H = 0$ *with probability 1.*

*Proof.* We will show that amongst all values of $\eta$, the value $P(P_i \leq p_i | P_j \leq p_j, H = \eta)$ is maximised when $\eta = 0$, from which the result follows.

From earlier considerations, we have

$$\begin{aligned} P(P_i \leq p_i | P_j \leq p_j, H = \eta) &= \frac{\iint_{|x|>z_i, |y|>z_j} N_{\binom{0}{\eta}, \begin{pmatrix} 1 & \rho \\ \rho & 1 \end{pmatrix}}(x, y) dx dy}{\int_{|y|>z_j} N_{(\eta,1)}(y) dy} \\ &\stackrel{\text{def}}{=} R_\eta(z_i, z_j) \end{aligned} \tag{35}$$

where $z_i = -\Phi^{-1}(p_i/2)$, $z_j = -\Phi^{-1}(p_j/2)$, and $N$ represents the normal *pdf* with the subscripted parameters. We may assume $\eta > 0$.

As $\eta$ increases from 0, the value of $R_\eta(z_i, z_j)$ (holding $(z_i, z_j)$ constant) tends to decrease to a minimum, then increase to an asymptote as $\eta \to \infty$ (figure 2).

We rewrite $R_\eta(z_i, z_j)$ as

$$\begin{aligned} R_\eta(z_i, z_j) &= \frac{\int_{|x|>z_i} \int_{|y|>z_j} \frac{1}{2\pi\sqrt{(1-\rho^2)}} exp\{\begin{pmatrix} x & y-\eta \end{pmatrix} \begin{pmatrix} 1 & \rho \\ \rho & 1 \end{pmatrix}^{-1} \begin{pmatrix} x \\ y-\eta \end{pmatrix}\} dy dx}{\int_{|y|>z_j} \frac{1}{\sqrt{2\pi}} exp\{-(y-\eta)^2\} dy} \\ &= \frac{\frac{1}{2\pi\sqrt{(1-\rho^2)}} \int_{|x|>z_i} \int_{|y|>z_j} exp\{-\frac{1}{2(1-\rho^2)}(x^2 - 2\rho x(y-\eta) + (y-\eta)^2)\} dy dx}{\frac{1}{\sqrt{2\pi}} \int_{|y|>z_j} exp\{-\frac{1}{2}(y-\eta)^2\} dy} \end{aligned} \tag{36}$$

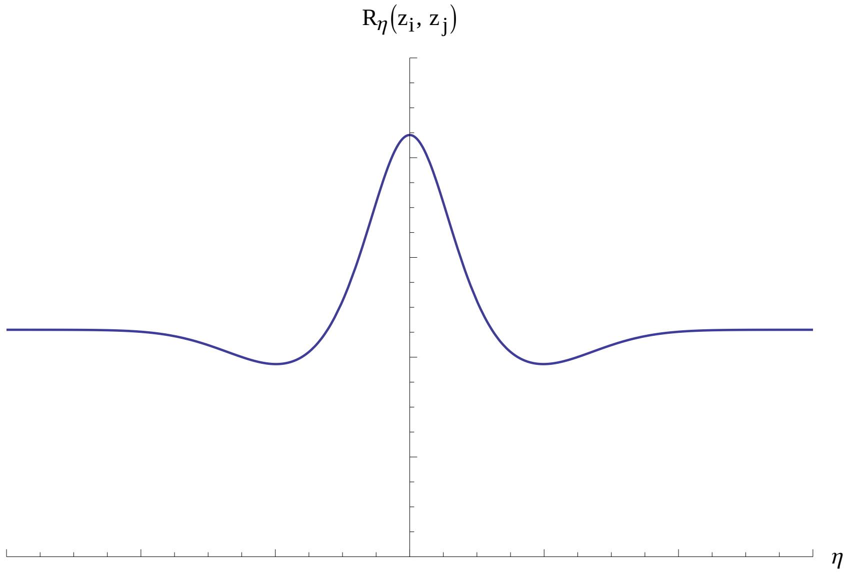


Figure 2: Plot of $R_\eta(z_i, z_j)$, or equivalently $Pr(P_i \leq p_i | P_j \leq p_j, H = \eta)$, as a function of $\eta$. The statement of the theorem is that this function is maximised at $\eta = 0$, which is seen in this plot. The function tends to drop to a minimum, then asymptote, as $\eta$ increases

The numerator may be rewritten as

$$
\begin{aligned}
&\frac{1}{2\pi\sqrt{(1-\rho^2)}} \int_{|x|>z_i} \int_{|y|>z_j} exp\{-\frac{1}{2(1-\rho^2)}((y-(\eta+x\rho))^2 + x^2(1-\rho^2))\} dydx \\
&= \int_{|x|>z_i} exp\{-\frac{1}{2}x^2\} \int_{|y|>z_j} \frac{1}{2\pi\sqrt{(1-\rho^2)}} exp\{-\frac{1}{2(1-\rho^2)}(y-(\eta+x\rho))^2\} dydx \\
&= \int_{|x|>z_i} \frac{exp\{-\frac{1}{2}x^2\}}{\sqrt{2\pi}} \big(\Phi(\frac{\eta+x\rho-z_j}{\sqrt{1-\rho^2}}) + \Phi(\frac{-\eta-x\rho-z_j}{\sqrt{1-\rho^2}})\big) dx \\
&= \int_{-\infty}^{-z_i} \frac{exp\{-\frac{1}{2}x^2\}}{\sqrt{2\pi}} \big(\Phi(\frac{\eta+x\rho-z_j}{\sqrt{1-\rho^2}}) + \Phi(\frac{\eta-x\rho-z_j}{\sqrt{1-\rho^2}}) \\
&\qquad\qquad + \Phi(\frac{-\eta+x\rho-z_j}{\sqrt{1-\rho^2}}) + \Phi(\frac{-\eta-x\rho-z_j}{\sqrt{1-\rho^2}})\big) dx \\
&\stackrel{\text{def}}{=} \int_{-\infty}^{-z_i} \frac{exp\{-\frac{1}{2}x^2\}}{\sqrt{2\pi}} K(x, z_j, \eta) dx
\end{aligned}
\tag{37}
$$

and the denominator simply as

$$\frac{1}{\sqrt{2\pi}}\int_{|y|>z_j} exp\{-\frac{1}{2}(y-\eta)^2\}dy = \Phi(-z_j+\eta)+\Phi(-z_j-\eta)$$
$$= B(z_j,\eta) \quad (38)$$

Since B is independent of $x$, we may simply include a factor of $1/B(z_j,\eta)$ in the integrand of 37.

If $z_i = 0$, then the numerator of 36 is an integral over the marginal of $y$, and hence the numerator and denominator are equal, so $R_\eta(0,z_j) = 1$. Hence

$$\begin{aligned} R_\eta(z_i,z_j) &= \int_{-\infty}^{-z_i} \frac{exp\{-\frac{1}{2}x^2\}}{\sqrt{2\pi}}\frac{K(x,z_j,\eta)}{B(z_j,\eta)}dx \\ &= 1-\int_{-z_i}^{0} \frac{exp\{-\frac{1}{2}x^2\}}{\sqrt{2\pi}}\frac{K(x,z_j,\eta)}{B(z_j,\eta)}dx \\ &= 1-\int_{0}^{z_i} \frac{exp\{-\frac{1}{2}x^2\}}{\sqrt{2\pi}}\frac{K(x,z_j,\eta)}{B(z_j,\eta)}dx \end{aligned}$$

given the symmetry of K in $x$. The problem then reduces to showing that

$$\int_0^{z_i} \frac{exp\{-\frac{1}{2}x^2\}}{\sqrt{2\pi}}\frac{K(x,z_j,\eta)}{B(z_j,\eta)}dx > \int_0^{z_i} \frac{exp\{-\frac{1}{2}x^2\}}{\sqrt{2\pi}}\frac{K(x,z_j,0)}{B(z_j,0)}dx \quad (39)$$

for all $\eta \neq 0$.

As $z_i \to \infty$, the numerator of 36 tends to 0 while the denominator does not change. Thus the above integral tends to 1 whatever the value of $\eta$.

For $z_i$ approaching 0, the ratio of the RHS integral to the LHS integral tends to the

ratio of the integrands at $x = 0$. This is equal to

$$\frac{K(0, z_j, \eta)B(z_j, 0)}{K(0, z_j, 0)B(z_j, \eta)} = \frac{(\Phi(\frac{\eta - z_j}{\sqrt{1-\rho^2}}) + \Phi(\frac{-\eta - z_j}{\sqrt{1-\rho^2}}))\Phi(z_j)}{(\Phi(\eta - z_j) + \Phi(-\eta + z_j))\Phi(\frac{-z_j}{\sqrt{1-\rho^2}})} \tag{40}$$

From the fact that $\Phi$ is convex on $\mathbb{R}^-$ and from $\Phi(x) = 1 - \Phi(-x)$, it is clear that if $b > a \geq 0$, $z > 0$ and $c_1$ and $c_2$ are such that $-z + a \leq c_1 \leq -z + b$, $-z - b \leq c_2 \leq -z + a$, then $\Phi'(c_1) > \Phi'(c_2)$. Thus $\Phi(-z + b) - \Phi(-z + a) > \Phi(-z - a) - \Phi(-z - b)$ and

$$\begin{aligned} &\Phi(\frac{-z_j + \eta}{\sqrt{1-\rho^2}}) - \Phi(\frac{-z_j}{\sqrt{1-\rho^2}} + \eta) > \Phi(\frac{-z_j}{\sqrt{1-\rho^2}} - \eta) - \Phi(\frac{-z_j - \eta}{\sqrt{1-\rho^2}}) \\ &\Leftrightarrow \Phi(\frac{-z_j + \eta}{\sqrt{1-\rho^2}}) + \Phi(\frac{-z_j - \eta}{\sqrt{1-\rho^2}}) > \Phi(\frac{-z_j}{\sqrt{1-\rho^2}} + \eta) + \Phi(\frac{-z_j}{\sqrt{1-\rho^2}} - \eta) \end{aligned} \tag{41}$$

We now define the function $\Phi^*(z) = \Phi(z + \eta)/\Phi(z)$, and note that the numerator of the derivative is

$$\begin{aligned} &\frac{1}{\sqrt{2\pi}} e^{-\frac{1}{2}(z+\eta)^2}\Phi(z) - \frac{1}{\sqrt{2\pi}} e^{-\frac{1}{2}z^2}\Phi(z + m) \\ &\propto \int_{-\infty}^{z} e^{-\frac{1}{2}(z+\eta)^2} e^{-\frac{1}{2}x^2} - e^{-\frac{1}{2}(x+\eta)^2} e^{-\frac{1}{2}z^2} dx \\ &= \int_{-\infty}^{z} e^{-\frac{1}{2}z^2} e^{-\frac{1}{2}x^2} e^{-\frac{1}{2}\eta^2} (e^{-\eta z} - e^{-\eta x}) dx \\ &< 0 \end{aligned} \tag{42}$$

as the integrand is always negative. Thus

$$\begin{aligned}
&\frac{\Phi(\frac{\eta-z_j}{\sqrt{1-\rho^2}})+\Phi(\frac{-\eta-z_j}{\sqrt{1-\rho^2}})}{\Phi(\frac{-z_j}{\sqrt{1-\rho^2}})} \\
&> \frac{\Phi(\frac{-z_j}{\sqrt{1-\rho^2}}+\eta)+\Phi(\frac{-z_j}{\sqrt{1-\rho^2}}-\eta)}{\Phi(\frac{-z_j}{\sqrt{1-\rho^2}})} \\
&= \frac{\Phi(\frac{-z_j}{\sqrt{1-\rho^2}}+\eta)}{\Phi(\frac{-z_j}{\sqrt{1-\rho^2}})}+\frac{\Phi(\frac{-z_j}{\sqrt{1-\rho^2}}-\eta)}{\Phi(\frac{-z_j}{\sqrt{1-\rho^2}})} \\
&> \frac{\Phi(-z_j+\eta)}{\Phi(-z_j)}+\frac{\Phi(-z_j-\eta)}{\Phi(-z_j)} \\
&= \frac{\Phi(-z_j+\eta)+\Phi(-z_j-\eta)}{\Phi(-z_j)}
\end{aligned} \tag{43}$$

and, rearranging, we see that expression 40 is greater than 1. Thus for sufficiently small $z_i$, inequality 39 holds.

We now show that the integrands on either side of 39 can be equal for at most one positive value of $x$, which we denote $x_0$. Since the integrals are equal as $z_i \to \infty$, they must in fact intersect exactly once. For $z_i < x_0$ the inequality obviously holds as the LHS integrand is strictly larger than the RHS integrand. For $z_i \geq x_0$, the integral of the LHS integrand from $z_i$ to $\infty$ is less than the integral of the RHS integrand over the same, so the integral of the LHS integrand from 0 to $z_i$ is again greater than the integral of the RHS integrand from 0 to $z_i$. This is shown in figure 3.

If the integrands are equal, then

$$\frac{K(x,z_j,\eta)}{K(x,z_j,0)}=\frac{B(z_j,\eta)}{B(z_j,0)}$$

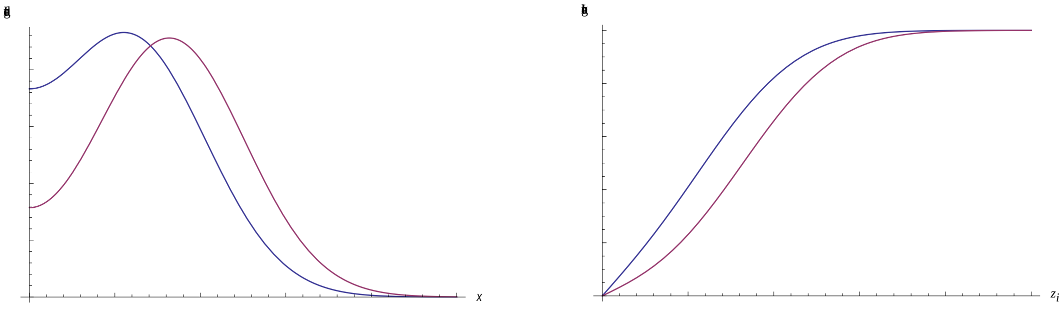


Figure 3: Plot of integrands (left) and integral (right) of expression 39, and integral, as functions of $x$ and $z_i$ respectively. We show that the integrands can only intersect once, as seen in the graph, so the integrals are never equal. This is seen on the right-hand plot.

so

$$\Leftrightarrow \frac{\Phi(\frac{\eta+x\rho-z_j}{\sqrt{1-\rho^2}}) + \Phi(\frac{\eta-x\rho-z_j}{\sqrt{1-\rho^2}}) + \Phi(\frac{-\eta+x\rho-z_j}{\sqrt{1-\rho^2}}) + \Phi(\frac{-\eta-x\rho-z_j}{\sqrt{1-\rho^2}})}{2\Phi(\frac{-x\rho-z_j}{\sqrt{1-\rho^2}}) + 2\Phi(\frac{x\rho-z_j}{\sqrt{1-\rho^2}})} = \frac{\Phi(\eta-z_j)+\Phi(-\eta-z_j)}{2\Phi(-z_j)} \tag{44}$$

From the convexity of $\Phi$ on $\mathbb{R}^-$ if $-x+m<0$ and the fact that the line between $(-x-m, \Phi(-x-m))$ and $(-x+m, \Phi(-x+m))$ lies strictly above $\Phi(y)$ for $y<0$, we have $(\Phi(-x+m)+\Phi(-x-m))/2 > \Phi(-x)$ so the RHS above is greater than 1. Considering the expression on the LHS as a function of $x$, we will show that it can take the required value $>1$ at most once on $\mathbb{R}^+$.

In analysing the LHS in isolation, the factor $\sqrt{1-\rho^2}$ merely scales $\eta$, and $z_j$, and the expression is to be proved for all $\eta, z_j > 0$, so we may ignore the factor in this case. Likewise, the factor $\rho/\sqrt{1-\rho^2}$ simply scales $x$, so we may ignore it as well.

If the LHS is to take some value $1+\epsilon/2$, then

$$\begin{aligned} &\Phi(-z_j-x+\eta)+\Phi(-z_j+x-\eta)+\Phi(-z_j+x-\eta)+\Phi(-z_j-x+\eta) \\ &-(2+\epsilon)(\Phi(-z_j-x)+\Phi(-z_j+x)) = 0 \end{aligned} \tag{45}$$

Consider the function

$$L_\epsilon(x,\eta) = (2+\epsilon)\Phi(x) - \Phi(x-\eta) - \Phi(x+\eta) \tag{46}$$

so 45 is equivalent to $L_\epsilon(x - z_j) + L_\epsilon(-x - z_j) = 0$. We have

$$\begin{aligned}\frac{\partial}{\partial x} L_\epsilon(x,\eta) \stackrel{\text{def}}{=} L'_\epsilon(x,\eta) &\propto (2+\epsilon)e^{-\frac{1}{2}x^2} - e^{-\frac{1}{2}(x-\eta)^2} - e^{-\frac{1}{2}(x+\eta)^2} \\ &= e^{-\frac{1}{2}x^2}((2+\epsilon) - e^{-\frac{1}{2}m^2} cosh(mx))\end{aligned} \tag{47}$$

so $L'_\epsilon(x,\eta)$ is zero only at two values $\pm x_\epsilon$. From the shape of the normal *pdf*, we have $L'_{epsilon}(0,\eta) > 0$ and because, for large enough $x$, we have

$$(2+\epsilon)e^{-\frac{1}{2}x^2} < e^{-\frac{1}{2}(x-\eta)^2} \tag{48}$$

$L'_\epsilon(x,\eta)$ must be asymptotically negative. Clearly $L'_\epsilon(x,\eta) = L'_\epsilon(-x,\eta)$, and $L'_\epsilon(x,\eta) \to 0$ as $x \to \infty$. Furthermore, the second derivative of $L_\epsilon(x,\eta)$ with respect to x (which we will call $L''_\epsilon(x,\eta)$) is given by

$$\begin{aligned}L''_\epsilon(x,\eta) &\propto (2+\epsilon)xe^{-\frac{1}{2}x^2} - (x+\eta)e^{-\frac{1}{2}(x+\eta)^2} - (x-\eta)e^{-\frac{1}{2}(x-\eta)^2} \\ &= e^{-\frac{1}{2}x^2}((2+\epsilon)x - e^{-\frac{1}{2}m^2}(2xcosh(mx) - 2msinh(-mx)))\end{aligned} \tag{49}$$

The curve $y = xcosh(mx) - msinh(-mx)$ can only intersect the line $y = xk/(2exp(-m^2/2))$ at one positive value of $x$, and given the antisymmetry of the curve and the line, $L''_\epsilon(x,\eta)$ has only three zeros. Given this and the earlier asymptotic properties, $L'_\epsilon(x,\eta)$ must be positive and maximal at 0, decrease monotonically to a negative minimum value, and increase monotonically thereafter. If we consider a superimposition of the curve transposed

left and transposed right the same amount, the transposed curves can only intersect at five points, one of which is $x = 0$.

The curves $L'_\epsilon(x - z_j, \eta)$ and $L'_\epsilon(-x - z_j, \eta) = L'_\epsilon(x + z_j, \eta)$ constitute two such transposed curves and thus

$$\frac{\partial}{\partial x}(L_\epsilon(x - z_j, \eta) + L_\epsilon(-x - z_j, \eta)) = L'_\epsilon(x - z_j, \eta) - L'_\epsilon(x + z_j, \eta) \tag{50}$$

can have at most two positive zeros. Because $L'_{epsilon}(x)$ is asymptotically increasing as $x \to \infty$, the difference $L'_\epsilon(x - z_j, \eta) - L'_\epsilon(x + z_j, \eta)$ is asymptotically negative, so the derivative of $L_\epsilon(x - z_j, \eta) + L_\epsilon(-x - z_j, \eta)$ is asymptotically increasing as $x \to \infty$. Writing

$$L_\epsilon(x, \eta) = 2\Phi(x) - \Phi(x - \eta) - \Phi(x + \eta) + \epsilon\Phi(x) \tag{51}$$

we see that as $x \to \infty$, $L_\epsilon(x, \eta) \to \epsilon\Phi(x) \to \epsilon$, so $L_\epsilon(x - z_j, \eta) + L_\epsilon(-x - z_j, \eta) \to 2\epsilon$. Because the derivative of $L_\epsilon(x - z_j, \eta) + L_\epsilon(-x - z_j, \eta)$ is asymptotically increasing toward 0, it must be asymptotically negative. Plots of $L_\epsilon$, $L'_\epsilon$ are shown in figure 4.

We now show that $L_\epsilon(x - z_j, \eta) + L_\epsilon(-x - z_j, \eta)$ is negative at $x = 0$. If it were to be positive, then

$$\begin{aligned} L_\epsilon(0 - z_j, \eta) + L_\epsilon(-0 - z_j, \eta) &> 0 \\ (2 + \epsilon)\Phi(x - zj) - \Phi(-z_j - \eta) - \Phi(-z_j + \eta) &> 0 \\ 1 + \frac{\epsilon}{2} &> \frac{\Phi(-z_j - \eta) + \Phi(-z_j + \eta)}{2\Phi(-zj)} \end{aligned} \tag{52}$$

Recalling, however, that the value of $1 + \epsilon/2$ of interest is given by the RHS of 44, which can be rewritten as

$$\frac{\Phi(\gamma(-z_j - \eta)) + \Phi(\gamma(-z_j + \eta))}{2\Phi(-\gamma zj)} \tag{53}$$

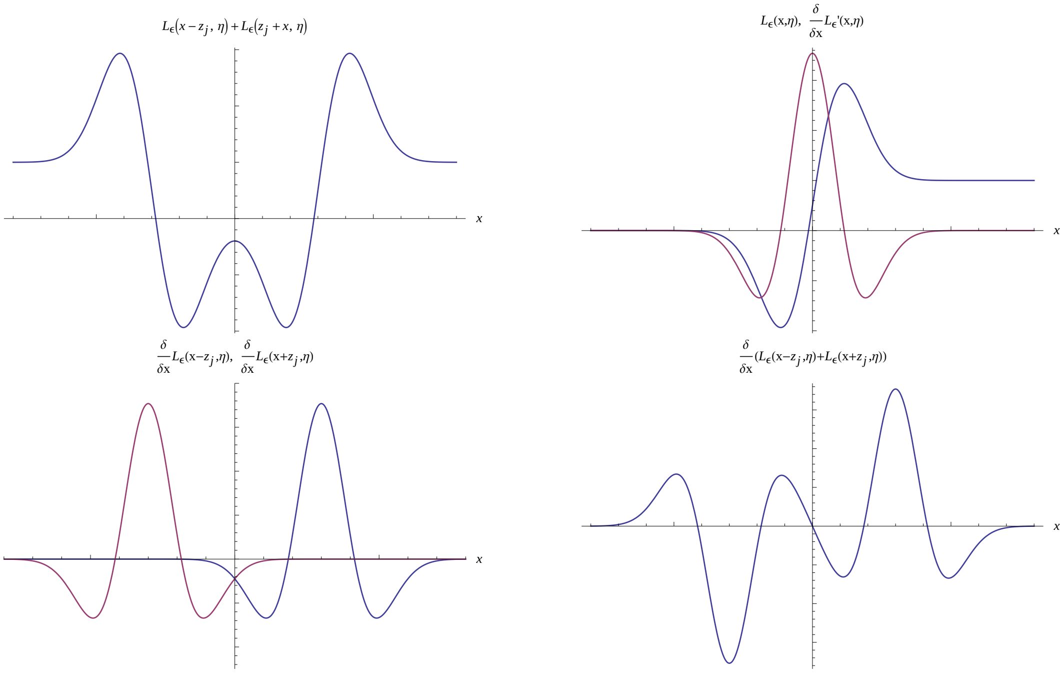


Figure 4: The top left plot shows $L_{epsilon}(x - z_j, \eta) + L_{epsilon}(x + z_j, \eta)$ as a function of $x$. We demonstrate that it is symmetric, asymptotically positive, has two stationary points for positive x, is negative at 0, and crosses zero once. These properties can be seen in this plot. The top right plot shows $L_{epsilon}(x, \eta)$ and its derivative with respect to $x$. The bottom right plot shows the derivative of $L_{epsilon}(x, \eta)$ transposed to the right and left by $z_j$. Note the limited number of points at which the two curves can cross. The bottom right plot shows the derivative with respect to $x$ of $L_{epsilon}(x - z_j, \eta) + L_{epsilon}(x + z_j, \eta)$

with $\gamma = \sqrt{1-\rho^2} < 1$, we see that we must have

$$\frac{\Phi(\gamma(-z_j - \eta)) + \Phi(\gamma(-z_j + \eta))}{2\Phi(-\gamma zj)} > \frac{\Phi(-z_j - \eta) + \Phi(-z_j + \eta)}{2\Phi(-zj)} \tag{54}$$

which is impossible as, given the shape of $\Phi$, the LHS is strictly increasing with $\gamma$ for $\gamma > 0$.

Thus for the value of $\epsilon$ of interest, $L_\epsilon(x - z_j, \eta) + L_\epsilon(-x - z_j, \eta)$ is negative at $x = 0$, asymptotically positive and decreasing as $x \to \infty$, and has as most two stationary points for $x > 0$. Consequently, it can cross any $y$ value between its height at 0 and its limit as $x \to \infty$ exactly once, and the result follows. □

# References


[1] Wood AR, Esko T, Yang J, Vedantam S, Pers TH, et al. (2014) Defining the role of common variation in the genomic and biological architecture of adult human height. Nature Genetics advance online publication: –.

[2] Benjamini Y, Hochberg Y (1995) Controlling the false discovery rate: a practical and powerful approach to multiple testing. Journal of the Royal Statistical Society, series B (methodological) 57: 289-300.

[3] Zaykin DV, Kozbur DO (2010) P-value based analysis for shared controls design in genome-wide association studies. Genetic Epidemiology 34: 725-738.

[4] Lin D, Sullivan PF (2009) Meta-analysis of genome-wide association studies with overlapping subjects. American Journal of Human Genetics 85: 862-872.
