# Supplementary figures and images for: A Pleiotropy-Informed Bayesian False Discovery Rate Adapted to a Shared Control Design Finds New Disease Associations From GWAS Summary Statistics
Source: PLoS Genet. 2015 Feb 6;11(2):e1004926. doi: 10.1371/journal.pgen.1004926 (PMC4450050; doi:10.1371/journal.pgen.1004926)

A. P values greater than  $1 \times 10^{-10}$

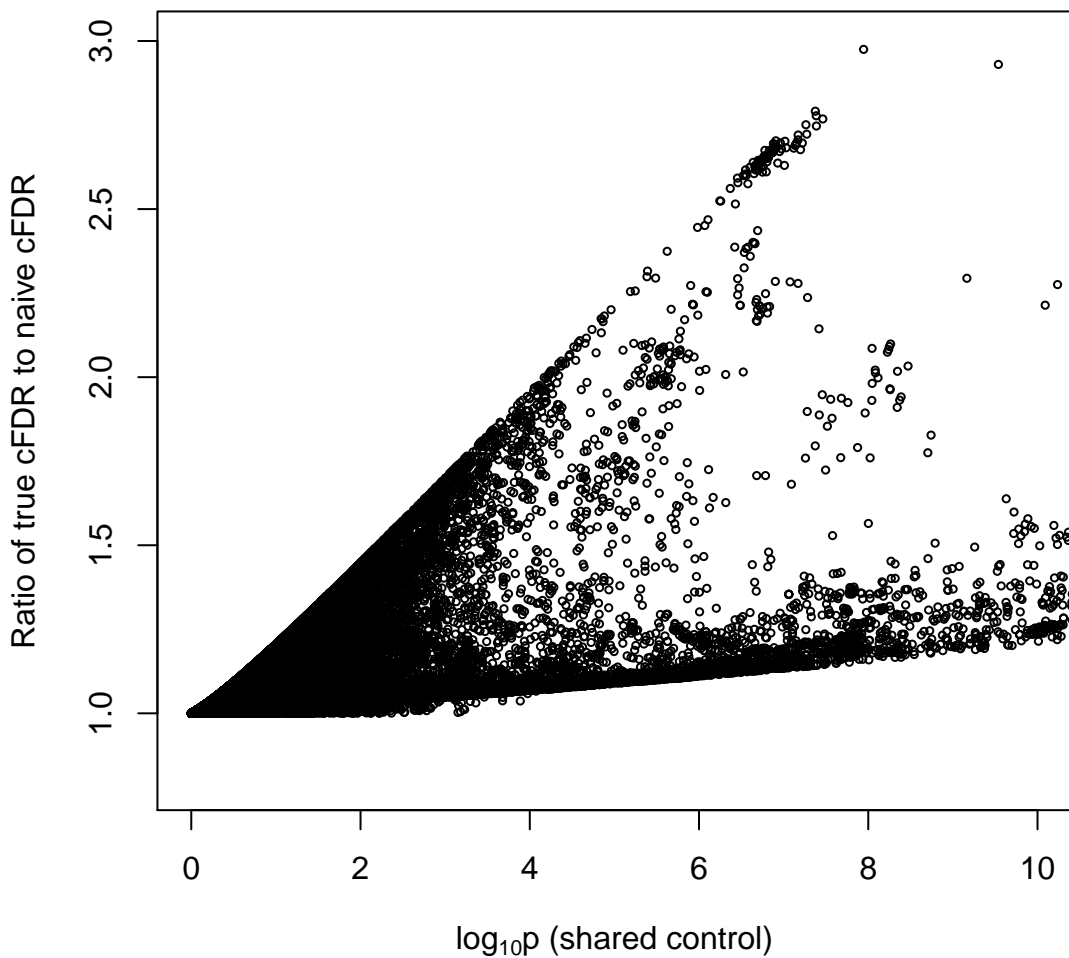

B. P values greater than  $1 \times 10^{-130}$

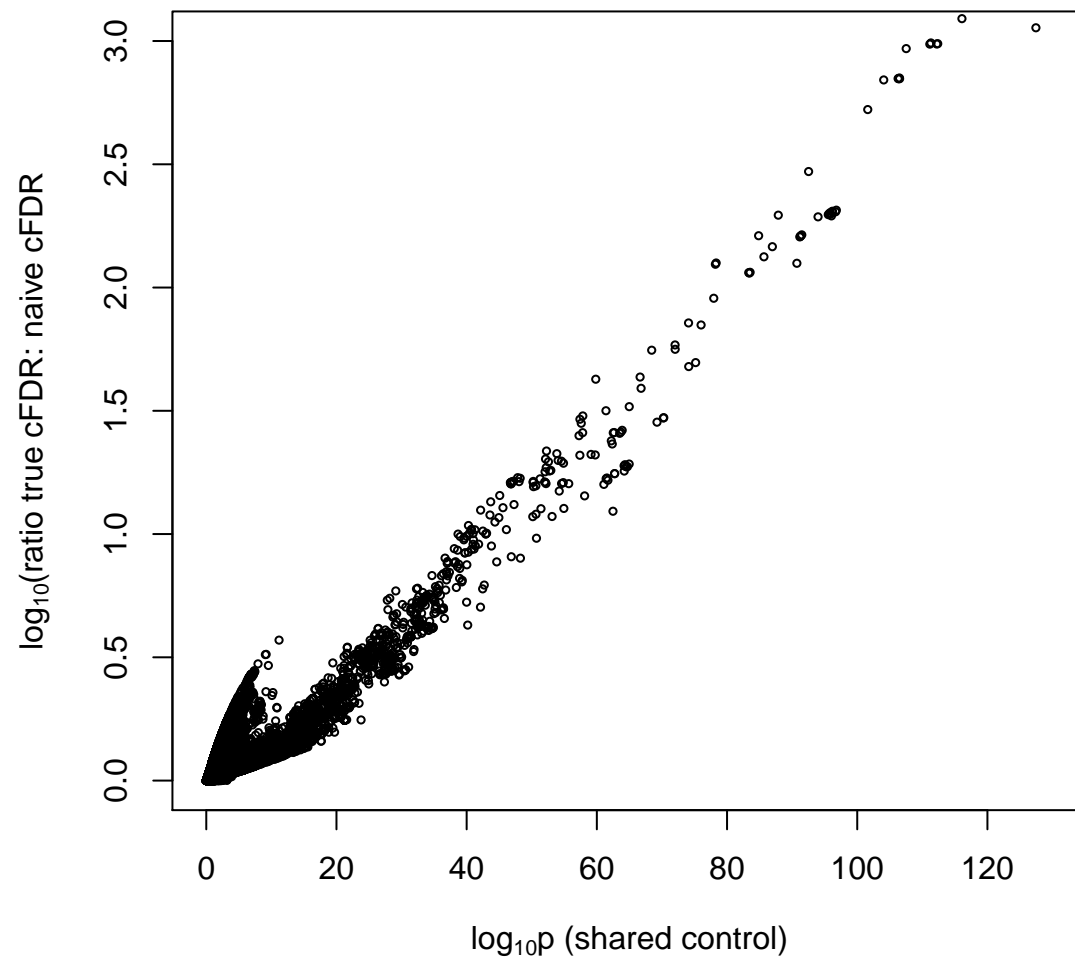

Supplement: S1 Fig — Plots A and B show the ratio between the true cFDR^ (computed using our method) to the’naive’ cFDR^ (computed by naively applying the existing split-control approach to shared-control data without adjustment) for a range of p values for the principal phenotype. The p values forming the x-coordinates were obtained from the shared-control design. The left-hand plot shows ratios of true to’naive’ cFDR for p values greater than 1 × 10?10, demonstrating 2–3 fold underestimation. The right-hand plot shows log-ratios of trueto’naive’ cFDR for smaller p values, demonstrating hundred- or thousand- fold underestimation. (PDF) [file pgen.1004926.s013.pdf]

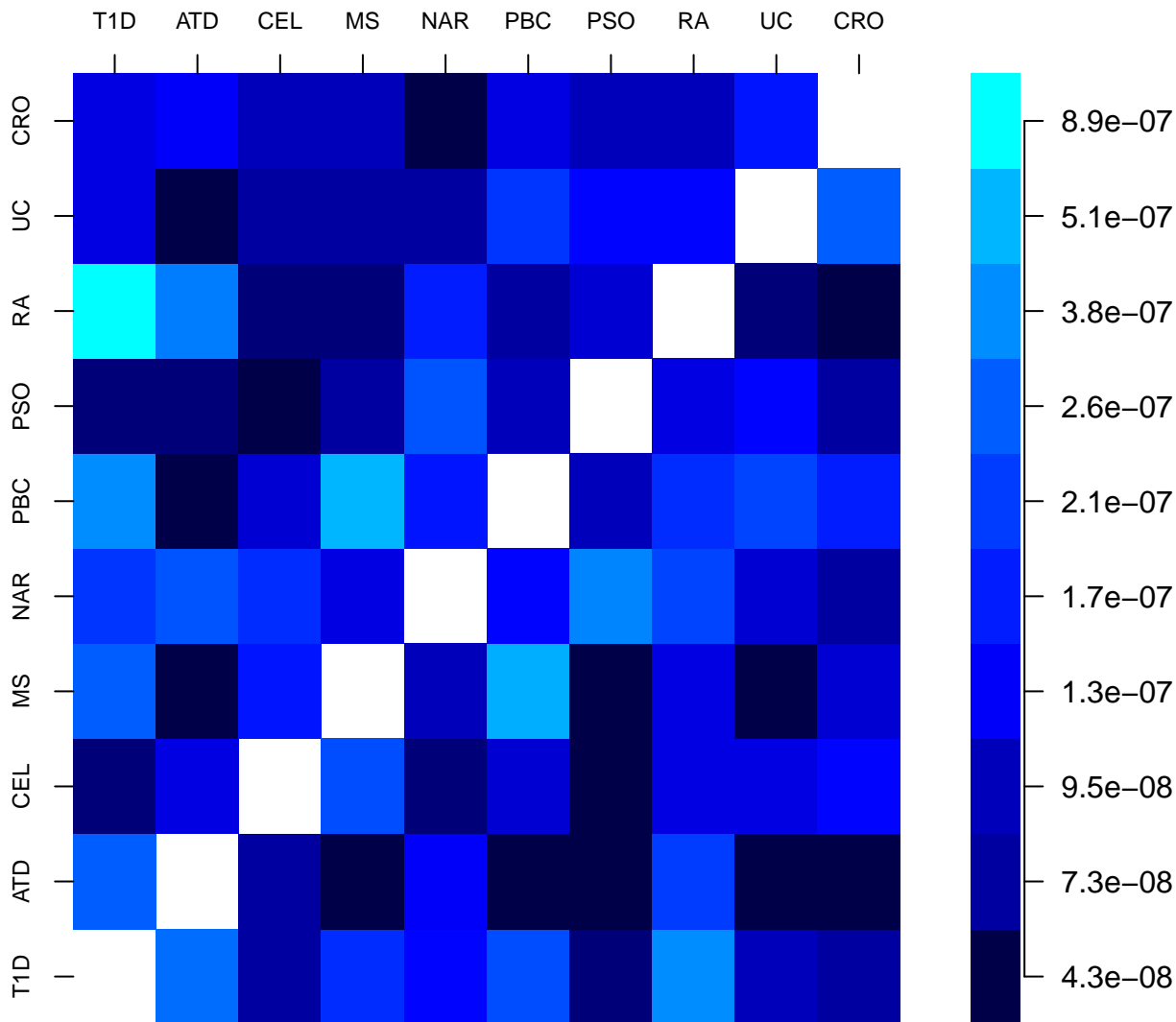

Supplement: S2 Fig — The colour for phenotype i (horizontal) and phenotype j (vertical) corresponds to the p-value cutoff for significance for phenotype i, given that a p-value cutoff for phenotype j is less than 5 × 10^−6^. (PDF) [file pgen.1004926.s014.pdf]

Frequency

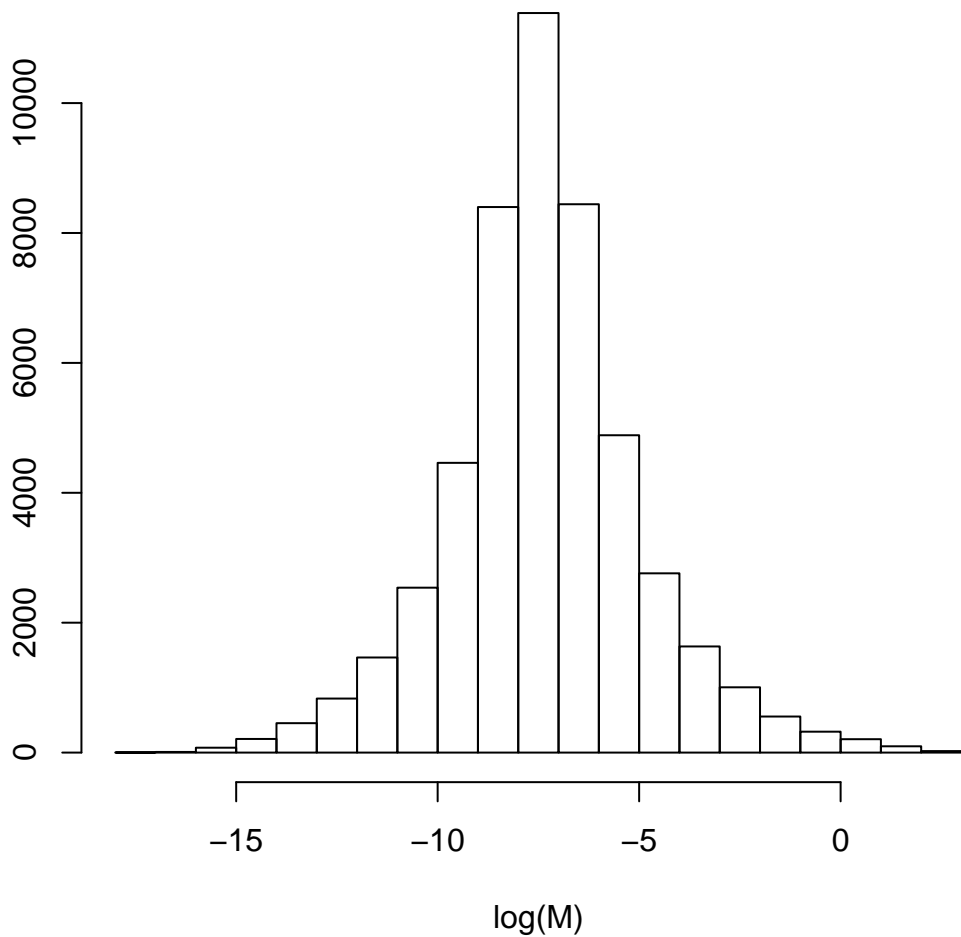

Supplement: S3 Fig — M is proportional to the variance of the log odds ratio from TDT data, defined as σ̂ 2 f(1−f)), where f is the minor allele frequency amongst null SNPs, and σ̂ is the standard error. Equating the median of M with a known expression for variance of the log odds ratio in a case-control study enables back-calculation of the effective number of cases and controls. This technique was used for computing the number of cases and controls in the T1D study, for which p values were obtained from a meta-analysis of case-control and TDT data. (PDF) [file pgen.1004926.s015.pdf]

T1D | ATD

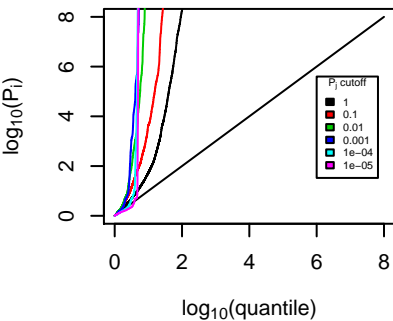

T1D | CEL

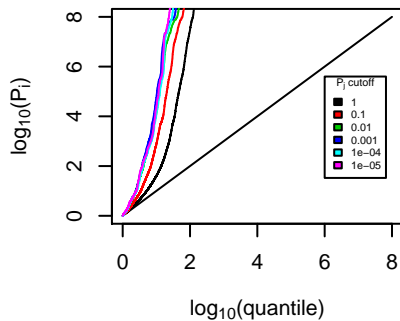

T1D | MS

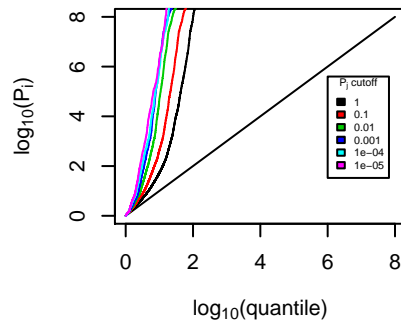

T1D | NAR

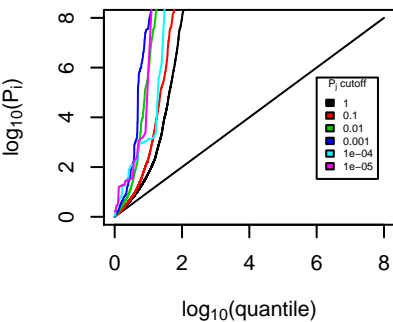

T1D | PBC

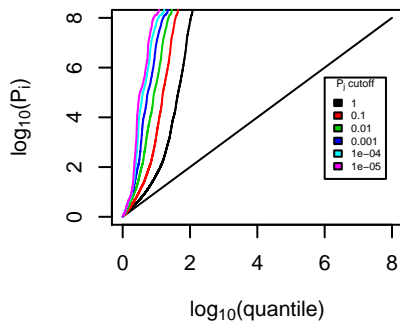

T1D | PSO

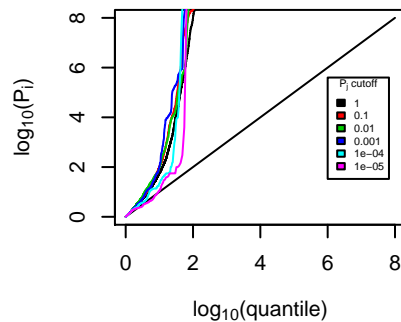

T1D | RA

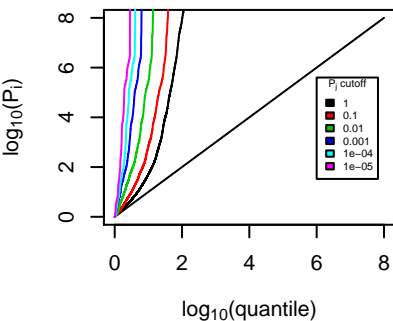

T1D | UC

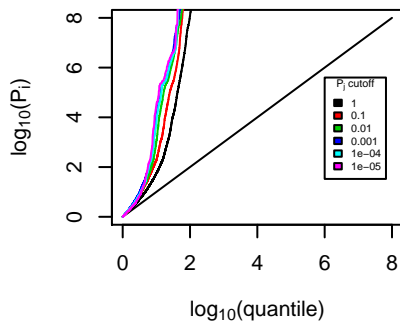

T1D | CRO

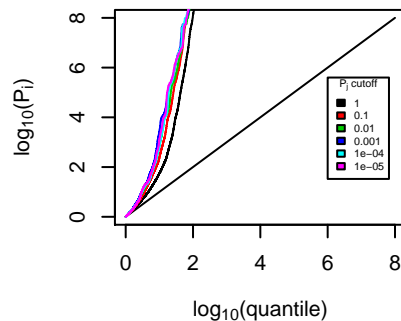

Supplement: S4 Fig — Y axes show log10(pi′); X axes show log quantile (rank) of p values in various sets of SNPs. Each colour corresponds to the Q-Q plot for p i amongst only SNPs such that p j is less than a certain cutoff, with the black line corresponding to the Q-Q plot for all SNPs. P values for the principal phenotype are adjusted for the effect of shared controls between studies. A leftward shift with decreasing p j cutoff indicates enrichment of SNP sets from conditioning on degrees of association with a conditional phenotype, probably due to pleiotropic effects between phenotypes. Because the studies used the ImmunoChip, which covers only potential autoimmune-associated regions, the black line also shows considerable enrichment compared to quantiles. S4 Fig. shows Q-Q plots with T1D (type 1 diabetes) as the principal phenotype (PDF) [file pgen.1004926.s016.pdf]

ATD | T1D

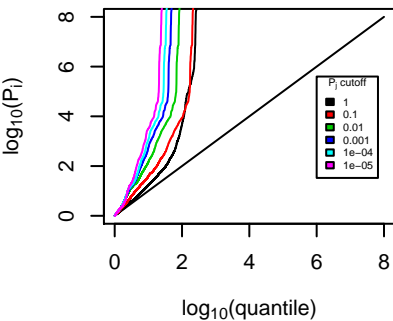

ATD | CEL

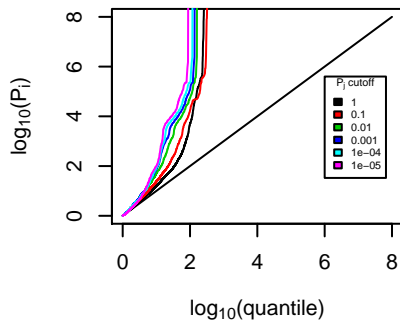

ATD | MS

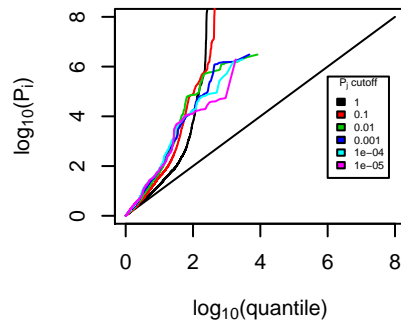

ATD | NAR

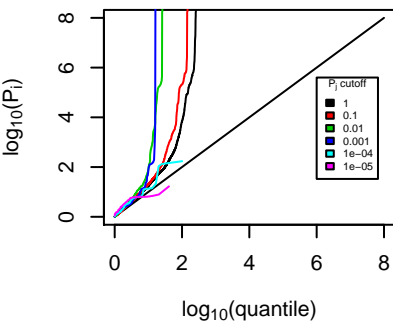

ATD | PBC

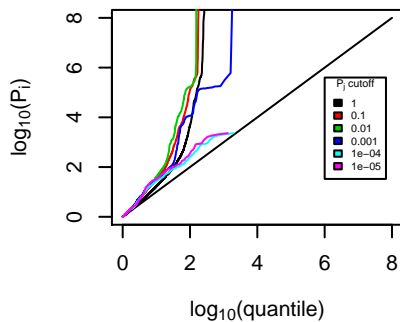

ATD | PSO

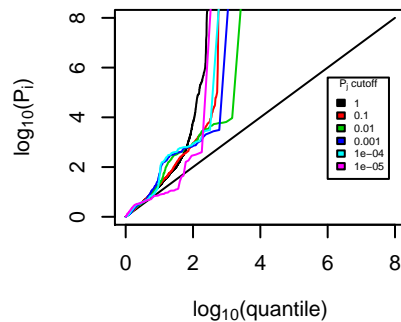

ATD | RA

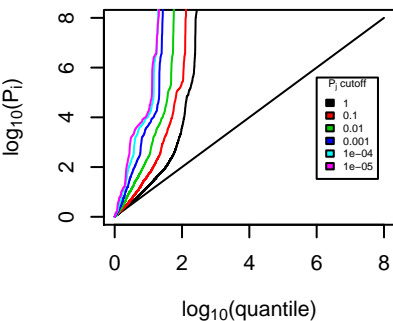

ATD | UC

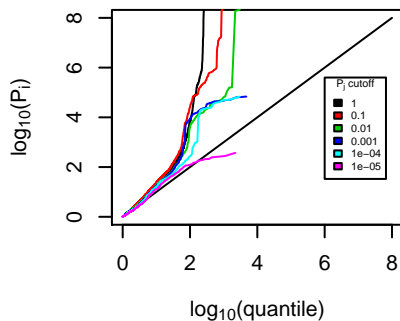

ATD | CRO

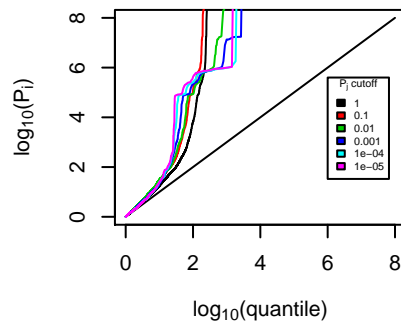

Supplement: S5 Fig — See legend for S4 Fig. (PDF) [file pgen.1004926.s017.pdf]

CEL | T1D

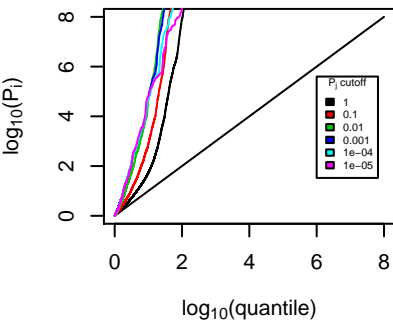

CEL | ATD

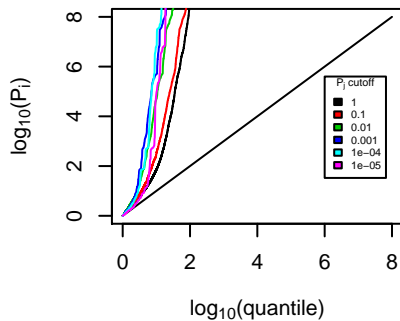

CEL | MS

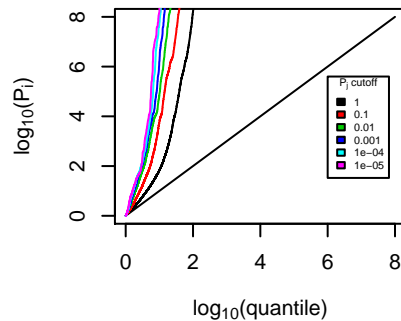

CEL | NAR

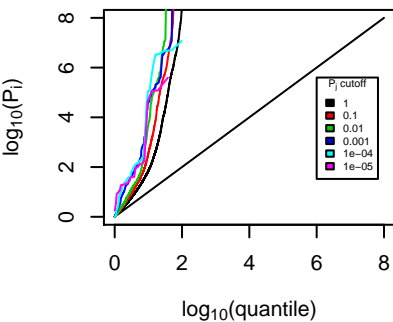

CEL | PBC

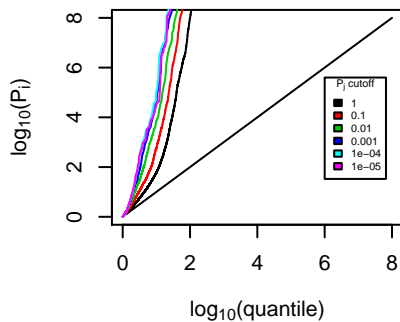

CEL | PSO

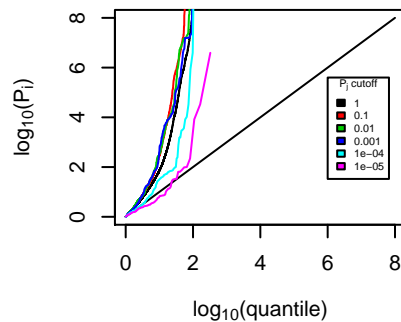

CEL | RA

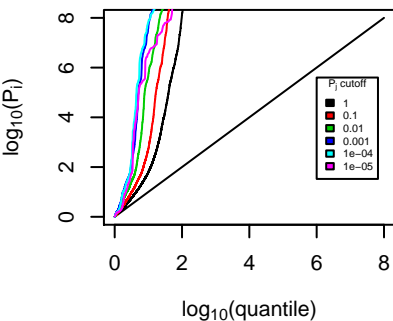

CEL | UC

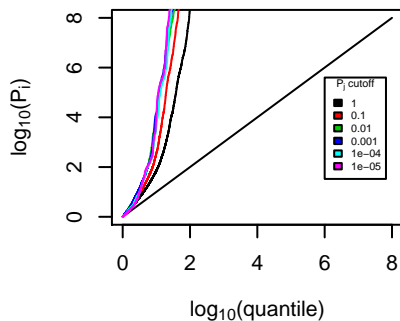

CEL | CRO

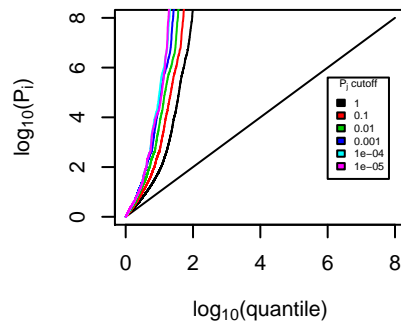

Supplement: S6 Fig — See legend for S4 Fig. (PDF) [file pgen.1004926.s018.pdf]

MS | T1D

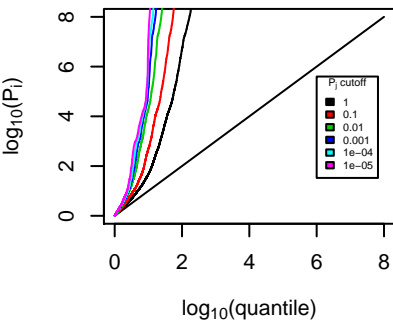

MS | ATD

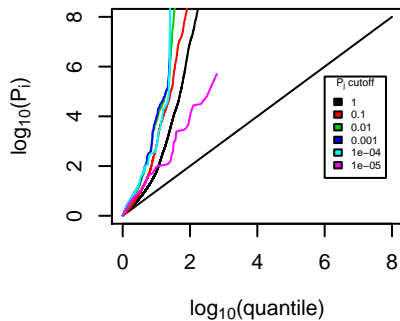

MS | CEL

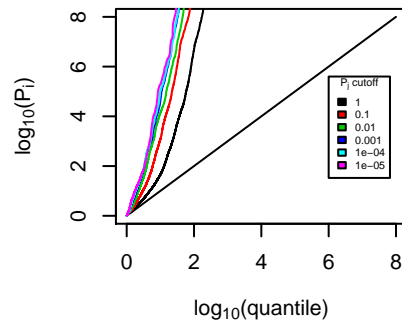

MS | NAR

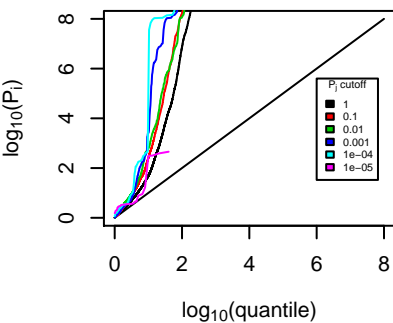

MS | PBC

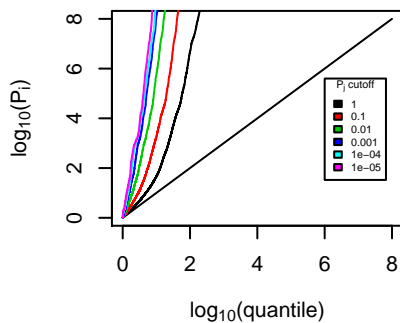

MS | PSO

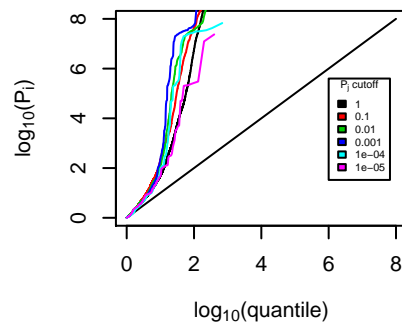

MS | RA

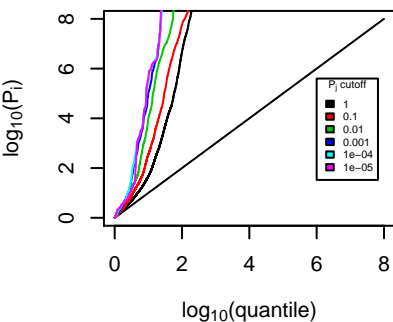

MS | UC

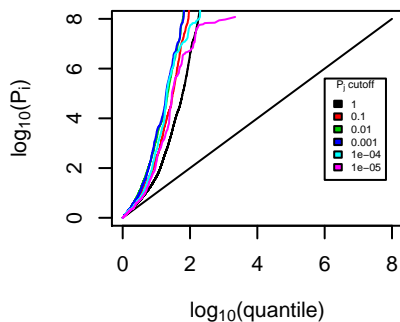

MS | CRO

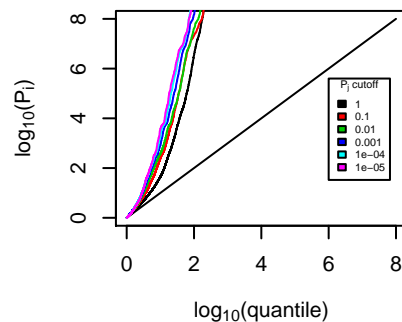

Supplement: S7 Fig — See legend for S4 Fig. (PDF) [file pgen.1004926.s019.pdf]

NAR | T1D

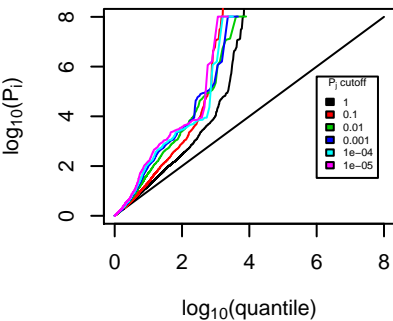

NAR | ATD

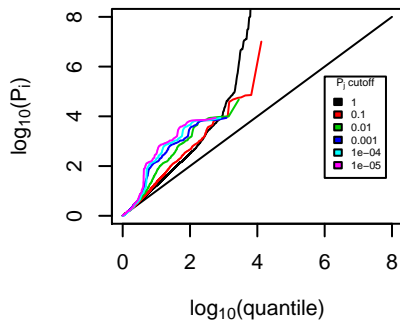

NAR | CEL

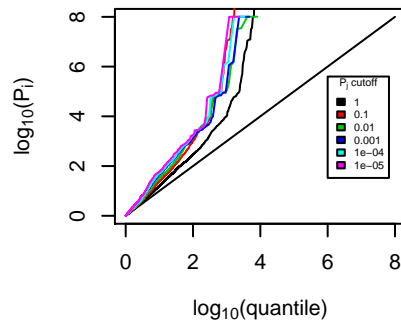

NAR | MS

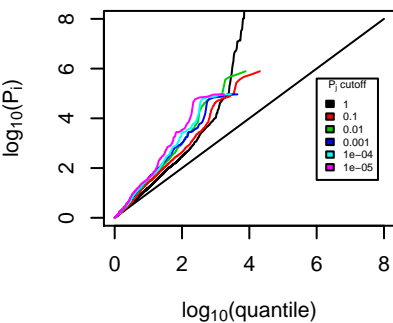

NAR | PBC

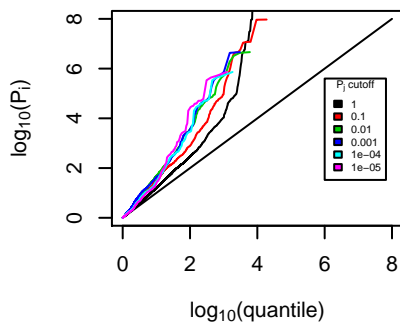

NAR | PSO

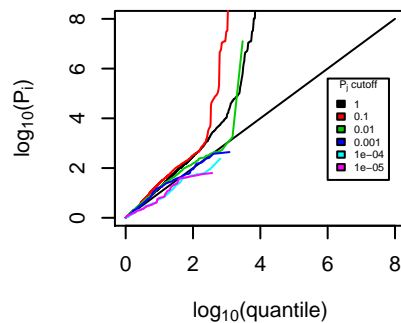

NAR | RA

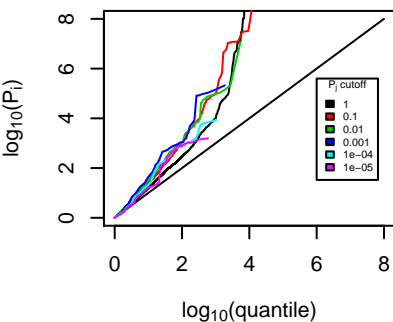

NAR | UC

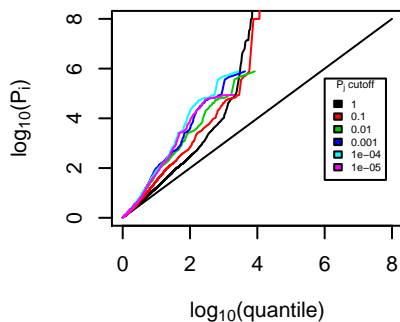

NAR | CRO

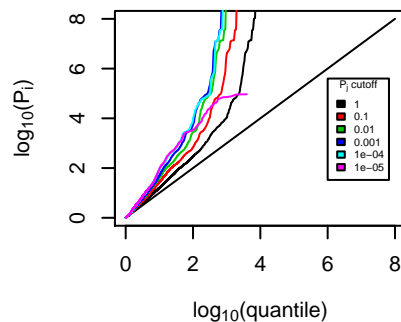

Supplement: S8 Fig — See legend for S4 Fig. (PDF) [file pgen.1004926.s020.pdf]

PBC | T1D

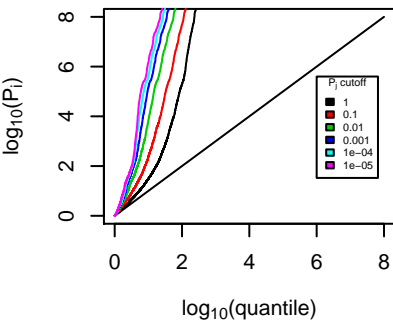

PBC | ATD

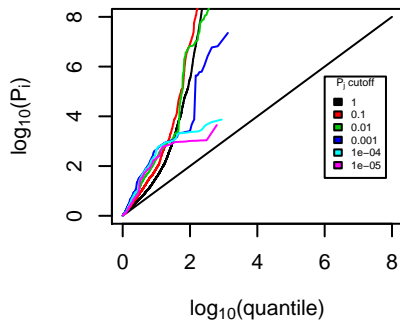

PBC | CEL

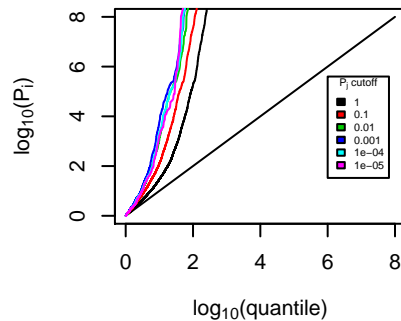

PBC | MS

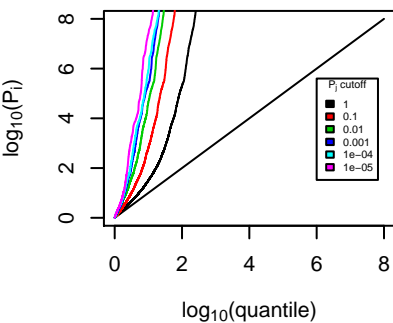

PBC | NAR

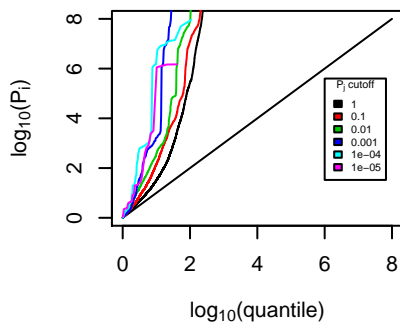

PBC | PSO

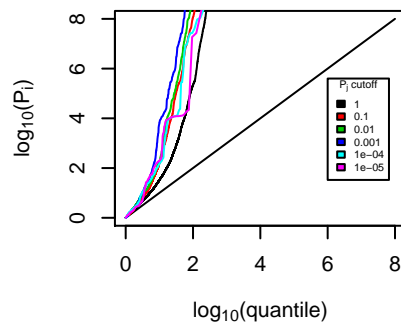

PBC | RA

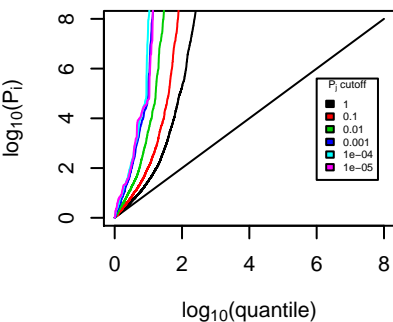

PBC | UC

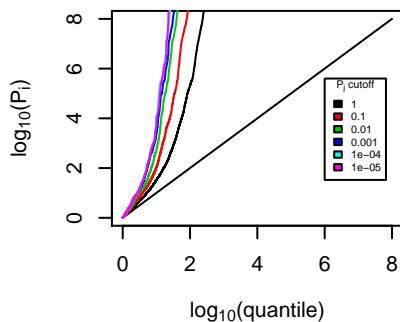

PBC | CRO

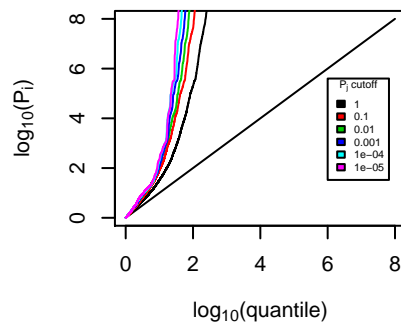

Supplement: S9 Fig — See legend for S4 Fig. (PDF) [file pgen.1004926.s021.pdf]

PSO | T1D

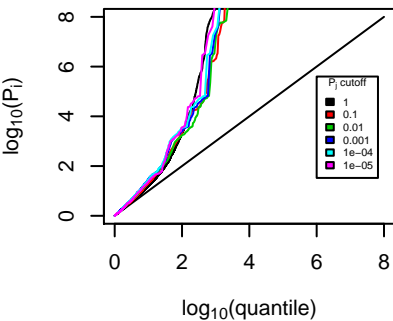

PSO | ATD

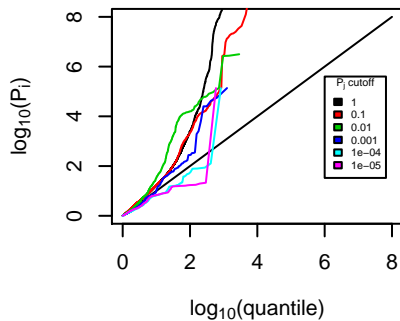

PSO | CEL

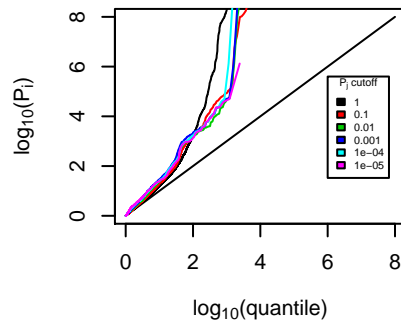

PSO | MS

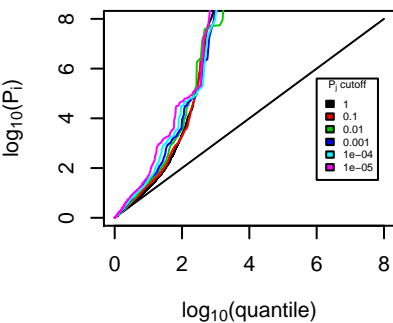

PSO | NAR

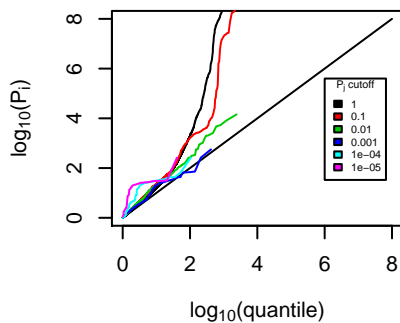

PSO | PBC

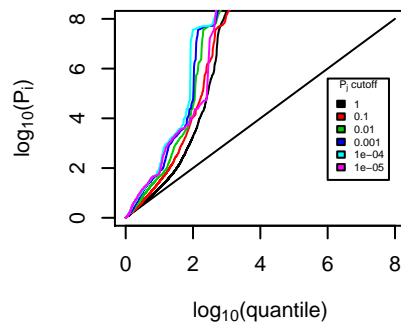

PSO | RA

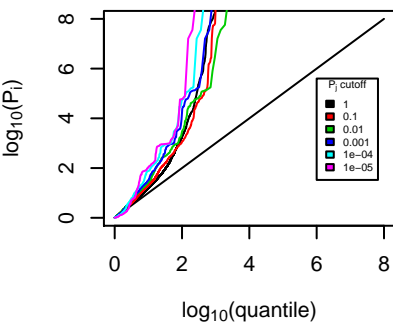

PSO | UC

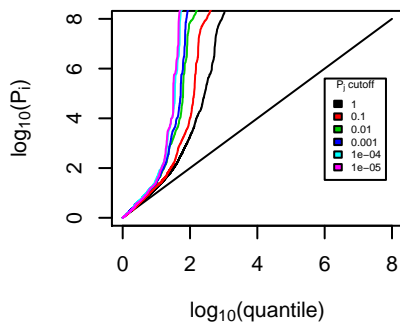

PSO | CRO

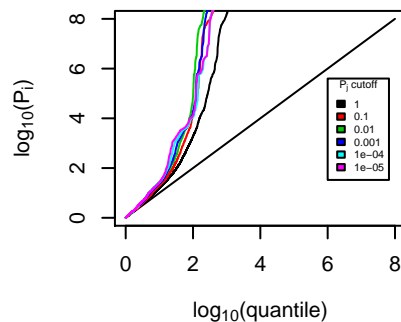

Supplement: S10 Fig — See legend for S4 Fig. (PDF) [file pgen.1004926.s022.pdf]

RA | T1D

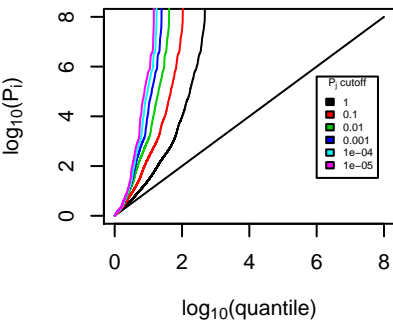

RA | ATD

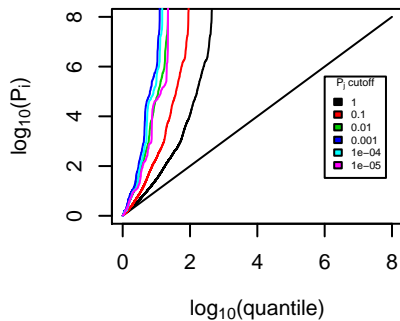

RA | CEL

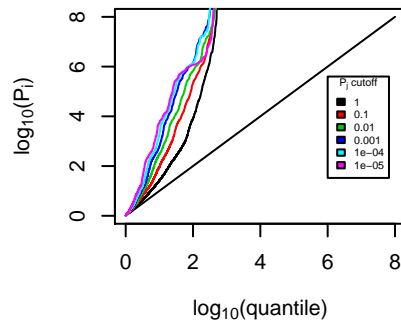

RA | MS

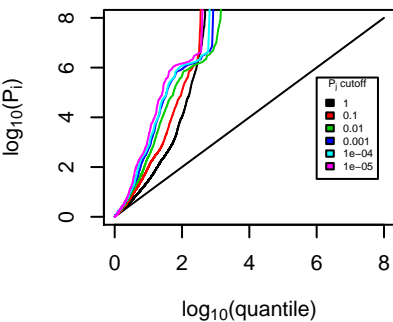

RA | NAR

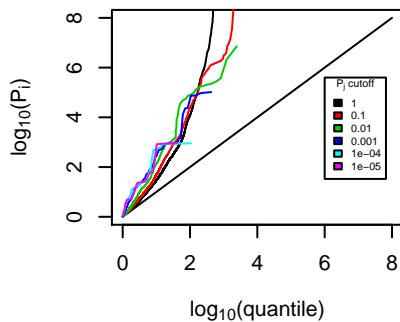

RA | PBC

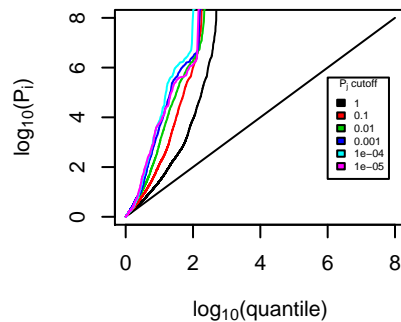

RA | PSO

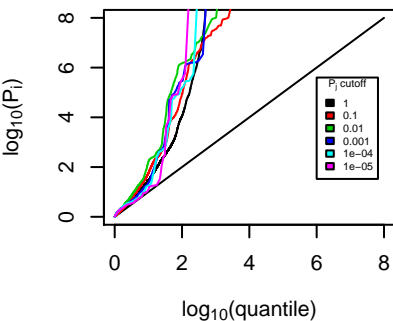

RA | UC

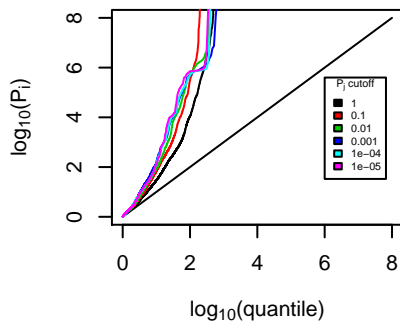

RA | CRO

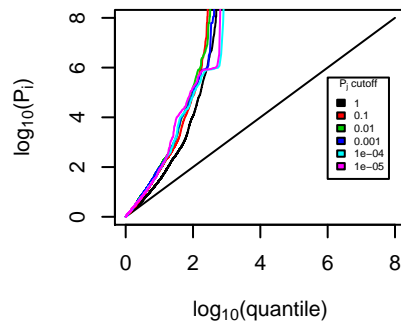

Supplement: S11 Fig — See legend for S4 Fig. (PDF) [file pgen.1004926.s023.pdf]

UC | T1D

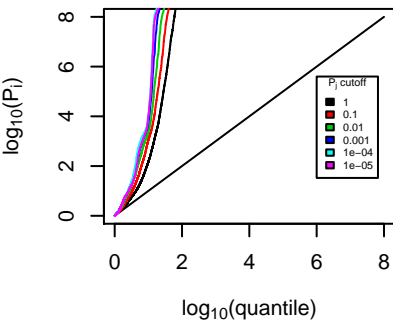

UC | ATD

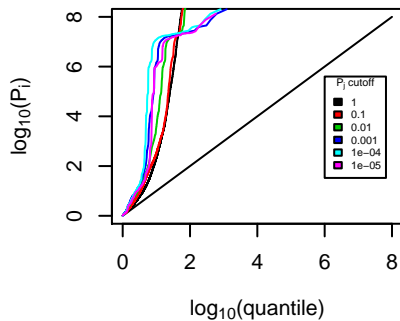

UC | CEL

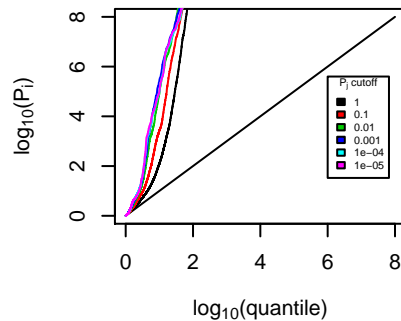

UC | MS

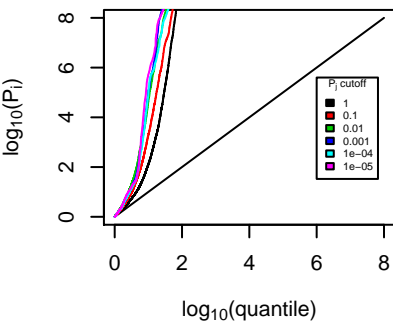

UC | NAR

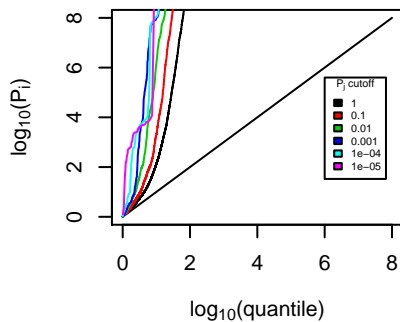

UC | PBC

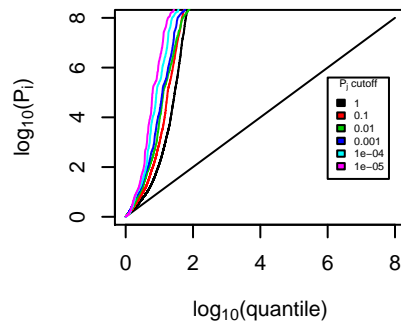

UC | PS

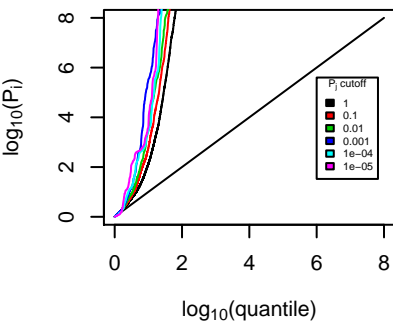

UC | RA

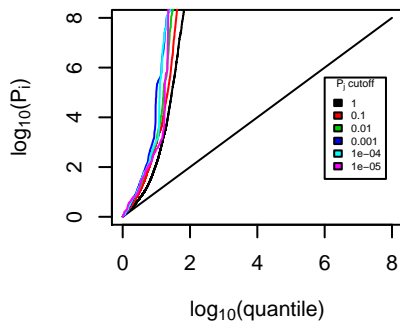

UC | CRO

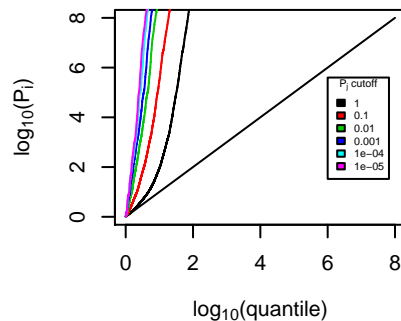

Supplement: S12 Fig — See legend for S4 Fig. (PDF) [file pgen.1004926.s024.pdf]

CRO | T1D

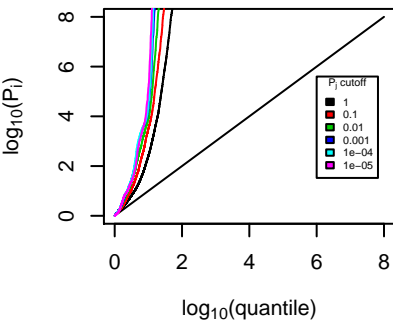

CRO | ATD

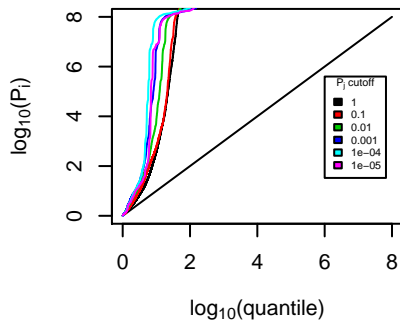

CRO | CEL

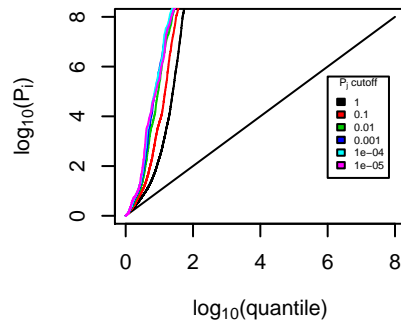

CRO | MS

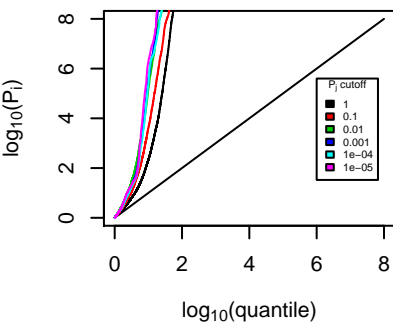

CRO | NAR

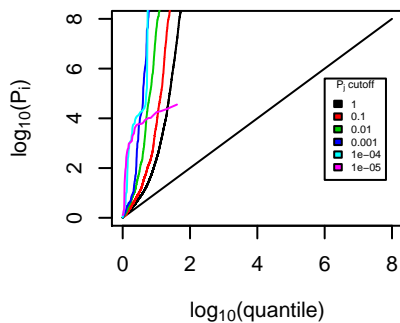

CRO | PBC

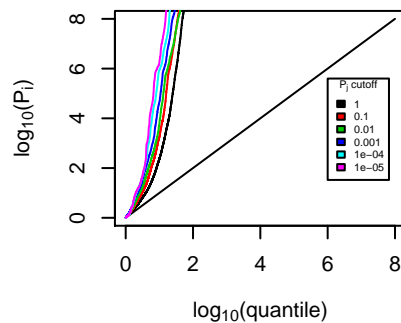

CRO | PSO

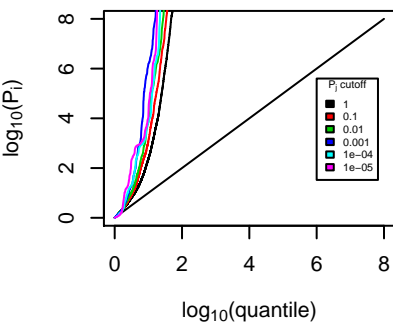

CRO | RA

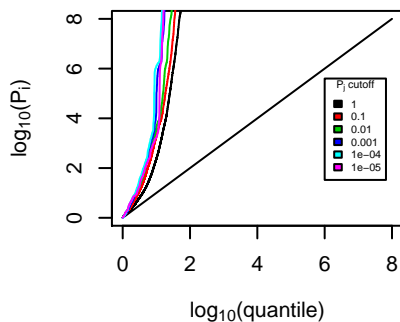

CRO | UC

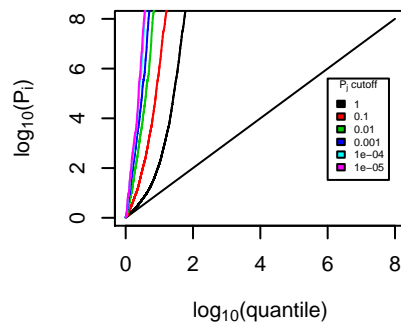

Supplement: S13 Fig — See legend for S4 Fig. (PDF) [file pgen.1004926.s025.pdf]
